# The proliferative activity levels of each immune cell population evaluated by mass cytometry are linked to the clinical phenotypes of systemic lupus erythematosus

**DOI:** 10.1093/intimm/dxac042

**Published:** 2022-08-23

**Authors:** Akiko Kajihara, Takayoshi Morita, Yasuhiro Kato, Hachiro Konaka, Teruaki Murakami, Yuta Yamaguchi, Shohei Koyama, Hyota Takamatsu, Masayuki Nishide, Yuichi Maeda, Akane Watanabe, Sumiyuki Nishida, Toru Hirano, Yoshihito Shima, Masashi Narazaki, Atsushi Kumanogoh

**Affiliations:** Department of Respiratory Medicine and Clinical Immunology, Graduate School of Medicine, Osaka University, 2-2 Yamadaoka, Suita, Osaka 565-0871, Japan; Laboratory of Immunopathology, World Premier International Immunology Frontier Research Center, Osaka University, 2-2 Yamadaoka, Suita, Osaka 565-0871, Japan; Department of Respiratory Medicine and Clinical Immunology, Graduate School of Medicine, Osaka University, 2-2 Yamadaoka, Suita, Osaka 565-0871, Japan; Laboratory of Immunopathology, World Premier International Immunology Frontier Research Center, Osaka University, 2-2 Yamadaoka, Suita, Osaka 565-0871, Japan; Department of Respiratory Medicine and Clinical Immunology, Graduate School of Medicine, Osaka University, 2-2 Yamadaoka, Suita, Osaka 565-0871, Japan; Laboratory of Immunopathology, World Premier International Immunology Frontier Research Center, Osaka University, 2-2 Yamadaoka, Suita, Osaka 565-0871, Japan; Department of Respiratory Medicine and Clinical Immunology, Graduate School of Medicine, Osaka University, 2-2 Yamadaoka, Suita, Osaka 565-0871, Japan; Department of General Medicine, Nippon Life Hospital, Public Interest Incorporated Foundation, 2-1-54 Enokojima, Osaka Nishi-ku, Osaka 550-0006, Japan; Department of Respiratory Medicine and Clinical Immunology, Graduate School of Medicine, Osaka University, 2-2 Yamadaoka, Suita, Osaka 565-0871, Japan; Laboratory of Immunopathology, World Premier International Immunology Frontier Research Center, Osaka University, 2-2 Yamadaoka, Suita, Osaka 565-0871, Japan; Department of Respiratory Medicine and Clinical Immunology, Graduate School of Medicine, Osaka University, 2-2 Yamadaoka, Suita, Osaka 565-0871, Japan; Laboratory of Immunopathology, World Premier International Immunology Frontier Research Center, Osaka University, 2-2 Yamadaoka, Suita, Osaka 565-0871, Japan; Department of Respiratory Medicine and Clinical Immunology, Graduate School of Medicine, Osaka University, 2-2 Yamadaoka, Suita, Osaka 565-0871, Japan; Laboratory of Immunopathology, World Premier International Immunology Frontier Research Center, Osaka University, 2-2 Yamadaoka, Suita, Osaka 565-0871, Japan; Department of Respiratory Medicine and Clinical Immunology, Graduate School of Medicine, Osaka University, 2-2 Yamadaoka, Suita, Osaka 565-0871, Japan; Laboratory of Immunopathology, World Premier International Immunology Frontier Research Center, Osaka University, 2-2 Yamadaoka, Suita, Osaka 565-0871, Japan; Department of Respiratory Medicine and Clinical Immunology, Graduate School of Medicine, Osaka University, 2-2 Yamadaoka, Suita, Osaka 565-0871, Japan; Laboratory of Immunopathology, World Premier International Immunology Frontier Research Center, Osaka University, 2-2 Yamadaoka, Suita, Osaka 565-0871, Japan; Department of Respiratory Medicine and Clinical Immunology, Graduate School of Medicine, Osaka University, 2-2 Yamadaoka, Suita, Osaka 565-0871, Japan; Integrated Frontier Research for Medical Science Division, Institute for Open and Transdisciplinary Research Initiatives (OTRI), Osaka University, 2-2 Yamadaoka, Suita, Osaka 565-0871, Japan; Department of Respiratory Medicine and Clinical Immunology, Graduate School of Medicine, Osaka University, 2-2 Yamadaoka, Suita, Osaka 565-0871, Japan; Laboratory of Thermotherapeutics for Vascular Dysfunction, Graduate School of Medicine, Osaka University, 2-2 Yamadaoka, Suita, Osaka 565-0871, Japan; Department of Respiratory Medicine and Clinical Immunology, Graduate School of Medicine, Osaka University, 2-2 Yamadaoka, Suita, Osaka 565-0871, Japan; Department of Respiratory Medicine and Clinical Immunology, Graduate School of Medicine, Osaka University, 2-2 Yamadaoka, Suita, Osaka 565-0871, Japan; Division of Rheumatology, Department of Internal Medicine, Nishinomiya Municipal Central Hospital, 8-24 Hayasidacho, Nishinomiya, Hyogo 663-8014, Japan; Department of Respiratory Medicine and Clinical Immunology, Graduate School of Medicine, Osaka University, 2-2 Yamadaoka, Suita, Osaka 565-0871, Japan; Laboratory of Thermotherapeutics for Vascular Dysfunction, Graduate School of Medicine, Osaka University, 2-2 Yamadaoka, Suita, Osaka 565-0871, Japan; Department of Respiratory Medicine and Clinical Immunology, Graduate School of Medicine, Osaka University, 2-2 Yamadaoka, Suita, Osaka 565-0871, Japan; Laboratory of Immunopathology, World Premier International Immunology Frontier Research Center, Osaka University, 2-2 Yamadaoka, Suita, Osaka 565-0871, Japan; Department of Advanced Clinical and Translational Immunology, Graduate School of Medicine, Osaka University, 2-2 Yamadaoka, Suita, Osaka 565-0871, Japan; Department of Respiratory Medicine and Clinical Immunology, Graduate School of Medicine, Osaka University, 2-2 Yamadaoka, Suita, Osaka 565-0871, Japan; Laboratory of Immunopathology, World Premier International Immunology Frontier Research Center, Osaka University, 2-2 Yamadaoka, Suita, Osaka 565-0871, Japan; Integrated Frontier Research for Medical Science Division, Institute for Open and Transdisciplinary Research Initiatives (OTRI), Osaka University, 2-2 Yamadaoka, Suita, Osaka 565-0871, Japan; Center for Infectious Diseases for Education and Research (CiDER), Osaka University, 2-2 Yamadaoka, Suita, Osaka 565-0871, Japan

**Keywords:** Ki-67, mass cytometry

## Abstract

Systemic lupus erythematosus (SLE) is a heterogeneous autoimmune disease, and many peripheral immune cell populations (ICPs) are thought to be altered according to the course of the disease. However, it is unclear which ICPs are associated with the clinical phenotypes of SLE. We analyzed peripheral blood mononuclear cells (PBMCs) of 28 SLE patients using mass cytometry and identified 30 ICPs. We determined the proliferative activity of ICPs by measuring the proportion of cells expressing specific markers and Ki-67 among CD45^+^ cells (Ki-67^+^ proportion). We observed an increased Ki-67^+^ proportion for many ICPs of SLE patients and examined the association between their Ki-67^+^ proportions and clinical findings. The Ki-67^+^ proportions of five ICPs [classical monocyte (cMo), effector memory CD8^+^ T cell (CD8Tem), CXCR5^−^ naive B cell (CXCR5^−^ nB), and CXCR5^−^ IgD^−^CD27^−^ B cell (CXCR5^−^ DNB)] were identified as clinically important factors. The SLE Disease Activity Index (SLEDAI) was positively correlated with cMo and plasma cells (PC). The titer of anti-DNA antibodies was positively correlated with cMo, CXCR5^−^ nB, and CXCR5^−^ DNB. The C4 level was negatively correlated with CXCR5^−^ DNB. The bioactivity of type I interferon was also positively correlated with these ICPs. Fever and renal involvement were associated with cMo. Rash was associated with CD8Tem and CXCR5^−^ DNB. On the basis of the proliferative activity among five ICPs, SLE patients can be classified into five clusters showing different SLE phenotypes. Evaluation of the proliferative activity in each ICP can be linked to the clinical phenotypes of individual SLE patients and help in the treatment strategy.

## Teaser

Immune-cell proliferative activity in the disease process of SLE

## Graphical Abstract

**Graphical Abstract:**
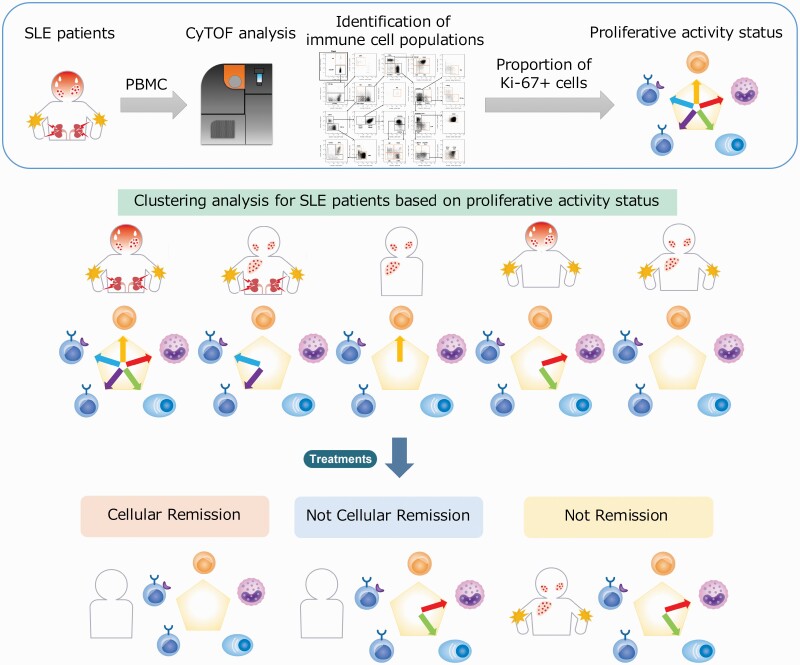

## Introduction

Systemic lupus erythematosus (SLE) is an autoimmune disease in which immune complexes are deposited in each organ and cause systemic inflammation (1–3). According to genome-wide association studies of SLE patients, abnormal immune system responses are important factors in SLE pathogenesis (4). As SLE is a heterogeneous disease, causing various organ disorders and relapsing during the long-term clinical course, the proportion and activity of immune cells responsible for this disease may differ in individual SLE patients and change dynamically over time. In immune cell populations (ICPs), B cells, plasmablasts (PB), and plasma cells (PC) are enriched within the peripheral blood of SLE patients, with B cells thought to play a central role in SLE pathology (5–7). Recently, CXCR5^−^ IgD^−^ CD27^−^ double negative B cells (CXCR5^−^ DNB) were shown to migrate to extrafollicular regions and are associated with lupus nephritis disease activity (8, 9). Type I interferon (IFN-I), a key factor in SLE pathogenesis, promotes differentiation into extrafollicular B cells, leading to the production of auto-antibodies (10, 11). Follicular helper CD4^+^ T cells (Tfh) and peripheral helper CD4^+^ T cells (Tph), which are enriched within SLE patients’ peripheral blood, also promote B cell activation (12, 13). In contrast, plasmacytoid dendritic cells (pDC) (a known IFN-I source) and monocytic cells are depleted in SLE patients (14, 15). This shows the ICP dysregulation during SLE. Most previous SLE studies have, however, focused on the dynamics of a single ICP, with few cluster analysis reports classifying individual SLE patients by circulating lymphocyte subset proportions (16).

In clinical practice, SLE disease activity and therapeutic efficacy are assessed using clinical findings such as anti-DNA antibody titers, complement levels, lymphocyte counts, and patient symptoms. However, those findings are not sufficient to understand SLE pathogenesis, and more detailed analyses of SLE patients’ ICP dynamics are required. Indeed, it is difficult to understand the pathogenesis of lymphocytopenia, often occurring in SLE patients, only by analysis of ICP proportions as identified by markers. For example, in the macrophages of SLE patients, IFN-I induces apoptosis, decreasing their cell count, yet inflammatory cytokine production is enhanced (17). To clarify the role of the ICPs in SLE pathology, it is necessary to assess not only the proportion but also the function and activity of each ICP identified.

The Ki-67 protein is expressed during the late G1, S, G2, and M phases of the cell cycle, whereas resting, non-cycling cells (G0 phase), lack its expression. During mitosis, the Ki-67 protein is essential in perichromosomal layer formation, a ribonucleoprotein sheath that coats condensed chromosomes, thus preventing mitotic chromosome aggregation (18). Because of the absence of Ki-67 in quiescent cells (G0 phase), this protein is widely used as a clinical tumor marker (19). Furthermore, Ki-67^+^ immune cells are known to infiltrate renal tissue in SLE patients with lupus nephritis, with the proportion of peripheral Ki-67^+^ natural killer (NK) cells positively correlated to Systemic Lupus Erythematosus Disease Activity Index (SLEDAI) scores (20, 21). With regards to B cells, peripheral CD11c^+^ B cells in SLE patients showed enhanced CD69, CD86, and Ki-67 expression, whereas the membrane expression of CXCR5 and CD21 was diminished, suggesting their activation outside of germinal centers (22). With regard to monocytes, human monocytes are traditionally considered non-proliferative, but recent studies have shown classical monocytes are anti-apoptotic and capable of proliferating *in vitro* (23). In inflammatory osteoarthritis synovial tissue, classical macrophages express higher levels of the proliferation marker Ki-67 (24). Despite reports of increased Ki-67^+^ cells in SLE and inflammatory diseases, there are few reports linking Ki-67^+^ cells of various ICPs to the clinical phenotype of SLE.

To understand the function and cellular activity of immune cells in autoimmune diseases, activated cell cytokine production and marker expression are often investigated, but measuring many cytokines simultaneously using mass cytometry is non-trivial. Instead, peripheral whole blood samples were stimulated with toll-like receptor (TLR) ligands and analyzed by mass cytometry simultaneously for surface marker expression, intracellular signaling protein activation state, and cytokine production. Subsets of NK and T cells were found to selectively induce NF-kB in response to TLR2 ligands (25). Peripheral whole blood from both healthy donor and rheumatoid arthritis (RA) patients were stimulated with tumor necrosis factor-α (TNF-α), and cells analyzed by mass cytometry. The signaling responses to exogenous TNF-α were greater in RA patients than in the normal donor (26). However, these approaches are applied to a focused cell population, and require *in vitro* stimulation. Multiple ICPs are thought to be involved in the pathogenesis of SLE, but the large number of ICP activity markers complicate the analysis. More comprehensive screening approaches are required to evaluate the cellular activity in each ICP. Ki-67 is broadly expressed in most ICPs with proliferative potential. Therefore, a search of Ki-67 expression, representing cell proliferative activity, may be useful to determine activated immune cells in diverse ICPs.

To investigate this possibility, we performed immunophenotyping of patients with new-onset and treated SLE using mass cytometry (cytometry by time-of-flight: CyTOF). After identifying ICPs with specific markers, we determined the proliferative activity of each ICP by measuring the proportion of cells expressing the specific markers and Ki-67 (Ki-67^+^ cells) among CD45^+^ cells. We then examined the association between proliferative activity and clinical phenotypes of individual SLE patients. On the basis of the proliferative activity of five ICPs showing different SLE phenotypes, SLE patients were clustered and patient immunodynamics with new-onset, flared, or steroid-tapered SLE before and after immunosuppressive treatment were investigated.

## Methods

### Human samples

A total of 59 blood samples, 28 SLE patients (five males, 23 females, mean age: 41.3 years) and 15 healthy donors (HDs) (six males, nine females, mean age: 37.4 years), were obtained at Osaka University Hospital. Among 28 SLE patients, 14 had new-onset SLE without prior treatments (new-onset SLE), six had relapses despite treatments (flared SLE: FL), three were recruited prior to starting treatment with belimumab, and five were tapering off corticosteroids (steroid-tapered SLE: ST). Blood sampling was performed before starting treatment in new-onset SLE and before changing treatment in flared SLE. Then, blood sampling was performed for eight new-onset patients, five flared patients, and three patients starting belimumab at 1–3 months or 5 years after the first sampling. All SLE patients were diagnosed as per the 1997 American College of Rheumatology (ACR) classification criteria for SLE (27). Disease activity was determined by SLEDAI scores (28). The patients’ backgrounds are shown in Table 1. SLE patients complicated with renal involvement were defined as patients with abnormal urinary test findings, such as urinary protein detection, pathological urinary casts, or elevated β-2 microglobulin levels. Each clinical phenotype of SLE in individual patients was retrospectively determined from the patients’ electronic medical records. Each symptom and laboratory data were obtained at the same time point as blood sampling. All SLE patients and HDs provided written informed consent in accordance with the Declaration of Helsinki.

**Table 1.** Characteristics of SLE patients

Patient	Age years	Sex	Fever	Arthritis	Rash	Renal Inv.	Serositis	Neural Inv.	ANA fold	C3 mg/dl	C4 mg/dl	aDNA Ab titer	Lym. /µl	Plt. ×10^4^/µl	SLEDAI	IFN-I O.D.
New-onset																
SLE1	51	F	−	+	+	+	−	−	640	22	2	300	939	5.7	19	0.832
SLE2	21	F	−	+	+	+	−	+	80	120	9	19	778	23.0	16	0.402
SLE3	37	F	−	−	+	+	+	−	2560	44	3	50	2830	26.2	20	0.457
SLE4	17	M	+	+	−	+	−	−		59	8	260		14.8	19	0.839
SLE5	65	F	+	+	+	−	−	+	1280	43	4	230	811	19.8	19	0.684
SLE6	19	F	+	−	+	+	−	−		54	7	87	761	10.8	8	0.929
SLE7	40	M	+	+	+	+	−	−	2560	43	2	120	519	3.8	27	0.701
SLE8	53	F	−	−	−	+	−	−		61	10	25	719	27.8	8	0.510
SLE9	82	M	+	+	−	+	−	+	160	76	8	44	900	16.7	25	0.327
SLE10	44	F	+	+	+	−	+	−	160	72	26	49	1449	23.6	13	0.515
SLE11	49	F	−	−	−	−	−	−	320	84	18	13		23.0	8	0.205
SLE12	69	F	+	+	+	−	+	−	1280	97	33	2.8	1062	28.6	11	1.019
SLE13	44	F	−	+	−	+	−	−		28	2	110		17.3	20	0.646
SLE14	49	F	−	−	−	−	−	−	640	64	4	19	509	11.4	12	0.518
Treated																
FL1	36	F	+	−	−	+	−	−	160	34	5	200	661	19.2	21	0.620
FL2	24	M	−	−	−	−	−	+	640	58	8	13	1730	18.0	12	0.655
FL3	21	F	+	+	−	−	−	+	640	66	14	2		21.0	19	0.216
FL4	37	F	−	−	−	+	−	−	640	45	7	300	821	0.4	18	0.356
FL5	34	F	−	−	−	−	−	−	N/A	80	19	5.6	450	3.7	8	0.265
FL6	22	F	+	−	−	+	−	−	N/A	98	18	2	432	0.5	19	0.155
BEL1	35	F	+	+	+	−	−	−	2560	37	3	160	1929	23.7	17	0.249
BEL2	41	F	−	−	+	−	−	−		44	3	N/A		18.9	4	0.492
BEL3	45	F	−	−	−	−	−	−	N/A	69	8	57	470	34.2	10	0.382
ST1	25	F	−	−	−	N/A	−	−	640	108	12	2	2574	26.4	16	0.145
ST2	29	F	−	−	−	+	−	−	1280	71	11	2		21.2	4	0.154
ST3	65	F	−	+	−	−	−	−		76	5	28	567	23.7	14	0.551
ST4	57	F	−	−	−	N/A	−	−	1280	75	13	48	742	29.1	8	0.262
ST5	47	F	−	+	−	−	−	−		63	8	3.2	540	35.1	4	0.295

Inv., involvement; ANA, anti-nuclear antibody; aDNA Ab, anti-DNA antibody; Lym., the number of lymphocytes; Plt., the number of platelets; SLEDAI, Systemic Lupus Erythematosus Disease Activity Index 2000; IFN-I, type I interferons; O.D., optical density; FL, flared; BEL, belimumab; ST, patients with tapered steroid; M, male; F, female; N/A, not available.

### Isolation and preservation of peripheral blood mononuclear cells and plasma

Blood samples were collected into heparin tubes (TERUMO, Tokyo, Japan). Plasma isolated from blood samples was frozen at ‐80°C. Peripheral blood mononuclear cells (PBMCs) were isolated by density centrifugation using Ficoll-Paque Plus (GE Healthcare BioSciences, Tokyo, Japan). PBMCs were cryopreserved in liquid nitrogen by CELLBANKER (Nippon Zenyaku Kogyo, Tokyo, Japan).

### Cell-based reporter assay for type I interferon bioactivity

Protocols for the measurement of IFN-I bioactivity using a cell-based reporter system were adapted from a previous study (29). HEK-Blue IFN-α/β cells were purchased from InvivoGen (San Diego, CA, USA). In this study, the bioactivity of IFN-I in plasma was measured.

### Measurement of cytokine and chemokine levels in plasma

IL-6, IL-8, and IP-10 (CXCL10) levels in the plasma of SLE patients or HDs were measured with a flow cytometer (BD FACS Canto II) using a Cytometric Bead Array Kit (BD, San Diego, CA, USA).

### Antibodies

Information on the antibodies used for CyTOF is shown in Supplementary Table S1. Some antibodies were labeled with metals using MAXPAR X8 Polymer labeling kits (Fluidigm, South San Francisco, CA, USA). Cisplatin containing isotopically enriched ^194^Pt was purchased from Fluidigm. Indium chloride containing 95% ^115^In and 5% ^113^In was purchased from Trace Sciences. Following previous methods, indium was conjugated to an anti-CD45 antibody (clone: HI30, BioLegend, San Diego, CA, USA) (30).

### Cell staining and measurement by CyTOF

One million cells of each sample were thawed. A total of 1 μM Cell-ID Cisplatin-^198^Pt (Fluidigm, South San Francisco, CA, USA) was added over five minutes at room temperature (RT). Human Fc Receptor Blocking Reagent (Miltenyi Biotec, Bergisch Gladbach, Germany) was added at a 1:25 dilution over 15 min at RT. To limit the batch effect, we barcoded each sample by combinations of six types of anti-CD45 antibodies over 15 min at RT before pooling the samples together. All barcoded samples were combined and stained with antibodies specific for surface markers for 15 min at RT. The samples were fixed with 1 ml of Fix/Perm Concentrate (eBioscience, San Diego, CA, USA) for 1 h at RT. They were then stained in permeabilization buffer with antibodies specific for intra-cellular markers for 30 min followed by secondary antibody staining for 30 min at RT. Then they were incubated in 1 ml of Maxper Fix and Perm Buffer (Fluidigm, South San Francisco, CA, USA) with 1 μM Cell-ID Intercalator-Ir (Fluidigm, South San Francisco, CA, USA) for 1 h on ice. The samples were then suspended in a total of 15% EQ Four Element Calibration Beads (Fluidigm, South San Francisco, CA, USA) with Cell Staining Buffer (Fluidigm, South San Francisco, CA, USA). CyTOF data were collected at an event rate of <1000 events/second by using a CyTOF Helios system (Fluidigm, South San Francisco, CA, USA). Raw FCS data were normalized by bead-based normalization in CyTOF software (version 6.7.1014; Fluidigm, South San Francisco, CA, USA).

### Mass cytometry data analysis

As a preprocessing step, residual normalization beads, doublets, and dead cells were excluded from normalized FCS data using FlowJo® 10.6.1. Each patient’s FCS data were de-barcoded by gating based on anti-CD45 antibody-conjugated metals staining patterns (Supplementary Figure S1). Finally, 30 ICPs with specific markers were identified by gating on biaxial scatter plots using FlowJo® 10.6.1 (Supplementary Figure S2). Abbreviations for 30 ICPs are shown in the the Abbreviation list shown after Supplementary Table S4. The median (maximum/minimum) cell count for total CD45^+^ cells was 33 325 (509 301/2540) in this analysis. We also evaluated the proliferative activity of each ICP by measuring the proportions of cells expressing specific markers and Ki-67 among CD45^+^ cells (Ki-67^+^ proportion) (Supplementary Figure S3). It should be noted that in most, CD14^+^ CD16^−^ classical monocytes (cMo), CD16^+^ non-classical monocytes (ncMo), CD14^−^ CD16^−^ CD11^+^ HLA-DR^+^ antigen-presenting cells (APC), PB, and PC, Ki-67 expression was observed in both SLE patients and HDs. Therefore, cells expressing Ki-67 at high levels in SLE patients compared with HDs were regarded as Ki-67^+^ cells in these ICPs (cMo, ncMo, APC, PB, and PC). The mean expression levels of TLR9 in pDCs was also evaluated. All CyTOF data were analyzed by using FlowJo® 10.6.1.

### Classification of SLE patients

All SLE patients were classified into six clusters by *k*-means clustering using R version 3.1.2 according to the proliferative activity in 12 ICPs including cMo, APC, CD4^−^ CD8^−^ double negative CD3^+^ T cells (dnCD3T), CD45RA^−^ CCR7^−^ effector memory CD8^+^ T cells (CD8Tem), other central memory CD4^+^ T cell (oCD4Tcm), CD45RA^−^ CTLA^−^4^+^ Foxp3^+^ effector regulatory CD4^+^ T cells (eTreg), CXCR5^−^ IgD^+^ CD27^−^ naive B cells (CXCR5^−^ nB), CXCR5^−^ IgD^+^ CD27^+^ non-switched memory B cells (CXCR5^−^ NSMB), CXCR5^−^ IgD^−^ CD27^+^ switched memory B cells (CXCR5^−^ SMB), CXCR5^−^ DNBs, PB, and PC related to clinical phenotypes of SLE (27). Each patient data set was plotted by scatterplot based on the data of component 1 and 2 calculated by principal component analysis using the Ki-67^+^ proportions of the same 12 ICPs as above correlated with the clinical phenotype of SLE. The influence of the proliferative activity level in 12 ICPs on clustering for SLE patients is shown as a vector on the scatter plot. As the first step in this clustering, six random reference points were selected in the scatterplot. Each patient was classified into six groups according to the most proximal reference point. As the second step, a center point (centroid) in each classified group was calculated. Each patient was reclassified into six groups according to the most proximal center point. By repeating this process many times, SLE patients were finally classified into six groups (*k*-means clustering). We determined the number of clusters to be six, as this stratified the largest number of SLE patients without creating groups consisting of a single SLE patient.

### Radar charts

To visualize the major proliferative activity status in SLE patients, we evaluated the Ki-67^+^ proportion of five ICPs (cMo, CD8Tem, CXCR5^−^ nB, CXCR5^−^ DNB, and PC) based on the effect size in each ICP in the clustering analysis, and calculated the fold changes in the Ki-67^+^ proportion of each ICP for individual SLE patients from the mean Ki-67^+^ proportion of each ICP in all HDs, and showed these fold change data as a radar chart.

### Statistical analysis

All statistical analyses were conducted using R version 3.1.2 and EZR version 1.29 (31). The non-parametric Mann–Whitney *U* test was used for comparisons between two groups. The Kruskal–Wallis test was used for comparisons among the three groups. The correlation between the clinical findings reflecting SLE disease activity and the Ki-67^+^ proportion of each ICP was determined using Spearman’s rank correlation coefficient (ρ). Multiple regression analysis and logistic regression analysis were used for multivariate analysis. Although a *P* value < 0.05 is considered statistically significant in general statistical tests, the *P* value was adjusted for the sample numbers using Bonferroni correction with statistical multiplicity considerations (when we evaluated the correlation between the Ki-67^+^ proportion of 30 ICPs and clinical phenotypes of SLE, a *P* value < 0.00167 was considered statistically significant).

### Ethics approval

This research was approved by the local ethics committees of Osaka University Hospital (18050).

## Results

### The proportions of cells expressing specific markers and Ki-67 among CD45^+^ cells (Ki-67^+^ proportion) of each ICP in SLE patients

**Fig. 1.**
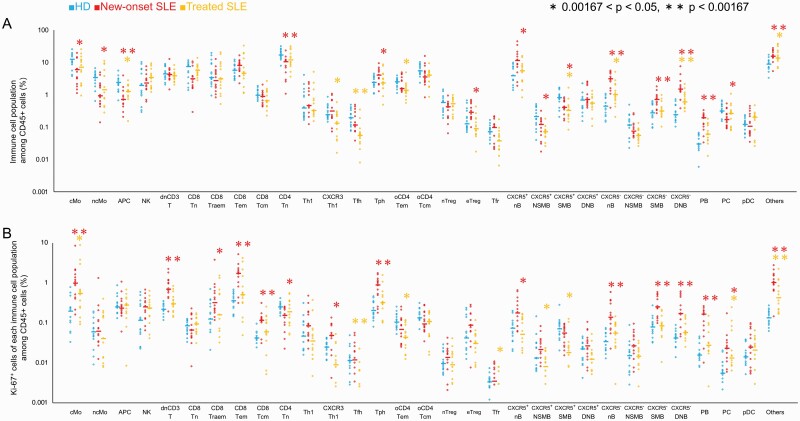
The proportions of cells expressing specific markers and Ki-67 among CD45^+^ cells (Ki-67^+^ proportion) of each ICP in SLE patients. (A) The proportion of ICP with specific surface markers among CD45^+^ cells in healthy donors (HDs) (*n* = 15, blue), patients with new-onset SLE (*n* = 14, red), and patients with treated SLE (*n* = 14, orange). (B) Proportions of cells expressing their surface markers and Ki-67 among CD45^+^ cells (Ki-67^+^ proportion). The Ki-67^+^ proportions of cMo, ncMo, APC, PB and PC shown based on proportions of cells expressing Ki-67 at high levels by comparison between SLE patients and HDs. The Mann–Whitney *U* test was used for comparisons between two groups. *0.00167 < *P* < 0.05, **P < 0.00167. See the Abbreviation list shown after Supplementary Table S4 for ICP abbreviations.

Peripheral ICPs in 28 SLE patients (14 new-onset and 14 treated) and 15 HDs were identified by gating on biaxial scatter plots (Supplementary Figure S2). Monocytes, APC, NK cells, CD4^+^ T cells, CD8^+^ T cells, B cells, and PC were detectable, and these sub-populations were further sub-divided into 30 ICPs with specific surface markers (see the Abbreviation list shown after Supplementary Table S4). The proportions of 30 ICPs among CD45^+^ cells in new-onset SLE patients, treated SLE patients, and HDs are shown in Fig. 1A. Significant increases were observed in ICPs including CXCR5^−^ nB, CXCR5^−^ SMB, CXCR5^−^ DNB, and PB in patients with new-onset SLE compared to HDs (median: CXCR5^−^ nB SLE 3.049%, HD 0.435%, *P* < 0.0001; CXCR5^−^ SMB SLE 0.716%, HD 0.267%, *P* = 0.0011; CXCR5^−^ DNB SLE 1.450%, HD 0.236%, *P* < 0.0001; PB SLE 0.189%, HD 0.0236%, *P* < 0.0001). Those of Tph and CXCR5^+^ IgD^+^ CD27^−^ naive B cell (CXCR5^+^ nB) slightly increased in patients with new-onset SLE (median: Tph SLE 3.957%, HD 2.370%, *P* = 0.0389; CXCR5^+^ nB SLE 11.494%, HD 3.829%, *P* = 0.0169). In contrast, those of APC, CD45RA^+^ CCR7^+^ naive CD4^+^ T cells (CD4Tn), CXCR5^+^ IgD^+^ CD27^+^ non-switched memory B cell (CXCR5^+^ NSMB), and CXCR5^+^ IgD^−^ CD27^+^ switched memory B cell (CXCR5^+^ SMB) were depleted significantly in patients with new-onset SLE compared to HDs (median: APC SLE 0.692%, HD 2.160%, *P* = 0.0015; CD4Tn SLE 10.395%, HD 16.524%, *P* = 0.0007; CXCR5^+^ NSMB 0.117%, HD 0.205%, *P* = 0.0309; CXCR5^+^ SMB SLE 0.404%, HD 0.807%, *P* = 0.0149). Those of cMo and ncMo were also slightly depleted in patients with new-onset SLE (median: cMo SLE 3.228%, HD 10.536%, *P* = 0.0088; ncMo SLE 0.901%, HD 3.323%, *P* = 0.0077). Increases in Tfh and PC proportions in SLE patients were not observed in our data (median: Tfh SLE 0.115%, HD 0.194%, *P* = 0.3682; PC SLE 0.165%, HD 0.309%, *P* = 0.0088).

Next, to determine the proliferative activity of ICPs, we measured the Ki-67^+^ proportion of 30 ICPs. For Ki-67^+^ cells in all PBMCs, the increase in Ki-67^+^ cells among CD45^+^ cells in patients with new-onset and treated SLE was determined as significant compared with HDs (median: new-onset SLE 8.787%, treated SLE 4.347%, HD 3.297%, new-onset SLE vs HD *P* < 0.0001, treated SLE vs HD *P* = 0.0018) (Supplementary Figure S4). The Ki-67^+^ proportions of each ICP among CD45^+^ cells are shown in Fig. 1B. The proliferative activity of ICPs including cMo, dnCD3T, CD8Tem, CD45^−^ CCR7^+^ central memory CD8^+^ T cells (CD8Tcm), Tph, CXCR5^−^ nB, CXCR5^−^ SMB, CXCR5^−^ DNB, and PB were significantly increased in patients with new-onset SLE compared with those in HDs (median: cMo SLE 0.975%, HD 0.194%, *P* = 0.0011; dnCD3T SLE 0.685%, HD 0.217%, *P* < 0.0001; CD8Tem SLE 1.667%, HD 0.349%, *P* = 0.0001; CD8Tcm SLE 0.115%, HD 0.041%, *P* = 0.0001; Tph SLE 0.863%, HD 0.196%, *P* = 0.0005; CXCR5^−^ nB SLE 0.137%, HD 0.033%, *P* = 0.0003; CXCR5^−^ SMB SLE 0.242%, HD 0.078%, *P* = 0.0002; CXCR5^−^ DNB SLE 0.166%, HD 0.041, *P* < 0.0001; PB SLE 0.158%, HD 0.0155%, *P* = 0.0002). The proliferative activity of CD45RA^+^ CCR7^−^ CD8^+^ T cells (CD8Traem), CD4Tn, CXCR3^+^ t-bet^+^ type 1 helper CD4^+^ T cells (CXCR3Th1), CXCR5^+^ IgD^+^ CD27^−^ naive B cell (CXCR5^+^ nB), and PC slightly increased in patients with new-onset SLE (median: CD8Traem SLE 0.317%, HD 0.121%, *P* = 0.0131; CD4Tn SLE 0.148%, HD 0.243%, *P* = 0.0044; CXCR3Th1 SLE 0.048, HD 0.024%, *P* = 0.0389; CXCR5^+^ nB SLE 0.174%, HD 0.071%, *P* = 0.0101; PC SLE 0.023%, HD 0.005, *P* = 0.0021). The proliferative activities of cMo and PC were also significantly elevated in patients with treated SLE (median: cMo treated SLE 0.535%, *P* = 0.0439; PC treated SLE 0.013, *P* = 0.0130).

The proliferative activities of some ICPs (dnCD3T, CD8Traem, CD8Tem, CD8Tcm) were found to be elevated in SLE patients compared to HDs (Fig. 1B), while there were no significant differences in the proportion of those ICPs among CD45^+^ cells (Fig. 1A). The proportions of cMo and PC in SLE patients were lower than those in HDs; however, their proliferative activities were elevated compared with HDs.

### Correlations between ICP proliferative activities and clinical findings in SLE patients

**Fig. 2.**
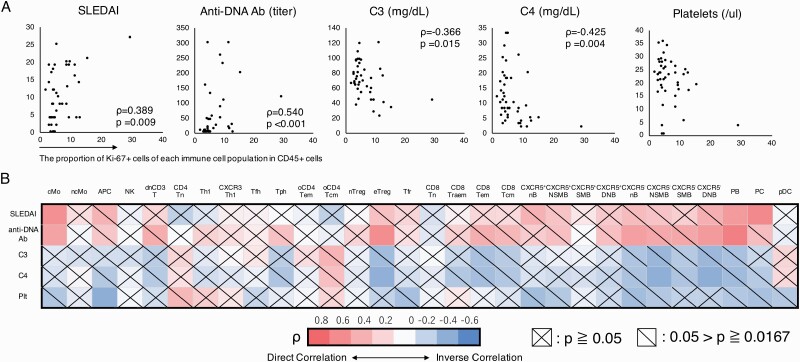
Correlations between ICP proliferative activities and clinical findings in SLE patients. (A) Correlations between proliferative activity based on Ki-67 expression between CD45^+^ cells and SLEDAI, anti-DNA antibody titer, C3 level, C4 level, and platelet count in SLE patients shown in scatter plots. (B) Heatmap of correlations between proliferative activity of each ICP and SLEDAI, anti-DNA antibody titer, C3 level, C4 level, and platelet count in SLE patients. Spearman’s rank correlation coefficients (ρ) were calculated for each evaluation. Colors in each box on the heatmap indicate the ρ value. SLEDAI: Systemic Lupus Erythematosus Disease Activity Index. See the Abbreviation list shown after Supplementary Table S4 for ICP abbreviations.

The correlation between the proliferative activity level of each ICP in SLE patients and clinical objective findings was examined with Spearman’s correlation test. First, the proportion of all Ki-67^+^ cells among CD45^+^ cells in SLE patients correlated with SLEDAI (ρ = 0.389, *P* = 0.009), titer of anti-DNA antibody (ρ = 0.540, *P* < 0.001), C3 level (ρ = ‐0.366, *P* = 0.015), and C4 level (ρ = ‐0.425, *P* = 0.004) but not platelet count (Fig. 2A).

Furthermore, correlations between the Ki-67^+^ proportion of each ICP and clinical findings were evaluated (Fig. 2B). SLEDAI was significantly positively correlated with the Ki-67^+^ proportion of cMo (ρ = 0.584, *P* = 0.00003), PB (ρ = 0.526, *P* = 0.00024), and PC (ρ = 0.619, *P* < 0.00001). The titer of anti-DNA antibody was significantly positively correlated with the Ki-67^+^ proportion of cMo (ρ = 0.484, *P* = 0.00101), eTreg (ρ = 0.598, *P* = 0.00002), CXCR5^−^ nB (ρ = 0.499, *P* = 0.00065), CXCR5^−^ DNB (ρ = 0.530, *P* = 0.00025), and PB (ρ = 0.664, *P* < 0.00001). The C4 level was significantly negatively correlated to the Ki-67^+^ proportion of eTreg (ρ = ‐0.503, *P* = 0.00050), CXCR5^−^ NSMB (ρ = ‐0.512, *P* = 0.00038), and CXCR5^−^ DNB (ρ = ‐0.518, *P* = 0.00032). The platelet count was significantly negatively correlated to the Ki-67^+^ proportion of APC (ρ = ‐0.519, *P* = 0.00031).

Based on the correlations among ICPs and clinical findings, multiple regression analysis was performed (Supplementary Table S2). The SLEDAI was positively correlated with the Ki-67^+^ proportion of PB (regression coefficient estimate 19.79, *P* = 0.0037). The titer of anti-DNA antibody and C4 level did not correlate significantly with the Ki-67^+^ proportions of all ICPs. For the C3 level and platelet count, multiple regression analysis was not performed as only two ICPs were found to correlate. These results suggested that the proliferative activity level of some ICPs, such as cMo, eTreg, CXCR5^−^ nB, CXCR5^−^ NSMB, CXCR5^−^ DNB, PB, and PC may reflect the SLEDAI, anti-DNA antibody titer, and C4 levels in SLE pathology.

### Correlations between the proliferative activity of each ICP and immunological factors in SLE patients

**Fig. 3.**
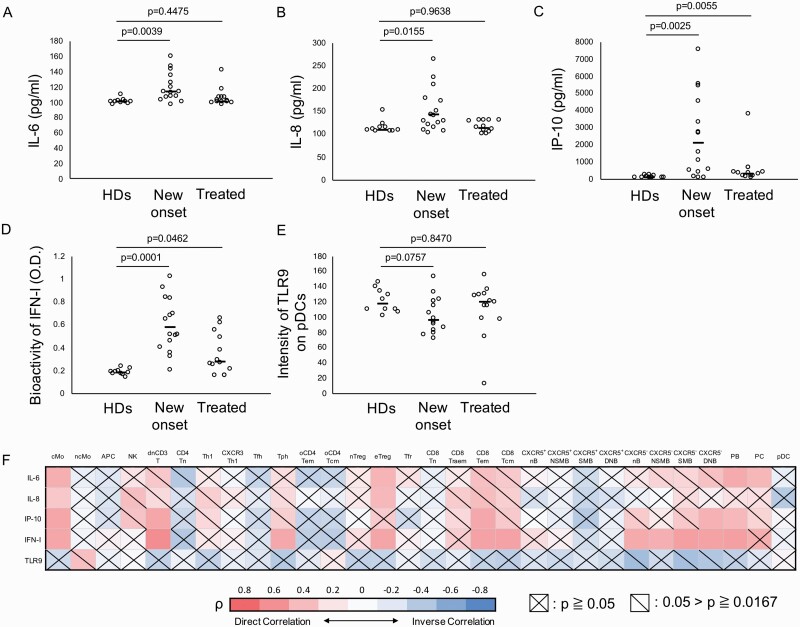
Correlations between the proliferative activity of each ICP and immunological factors in SLE patients. (A–C) IL-6, IL-8, and IP-10 (CXCL10) levels in plasma from SLE patients and HDs. (D) Bioactivity of IFN-I in plasma from SLE patients and HDs. (E) The intensity of TLR9 expression on pDC in SLE patients and HDs. (F) The correlations between the proliferative activity of each ICP and IL-6, IL-8, IP-10, IFN-I bioactivity and TLR9 expression intensity on pDC in SLE patients were shown as a heatmap. (A–E) The Kruskal–Wallis test was used for comparisons among three groups. (F) Spearman’s rank correlation coefficients (ρ) were calculated. *0.00167 < *P* < 0.05, **P < 0.00167. See the Abbreviation list shown after Supplementary Table S4 for ICP abbreviations.

To assess the role of cytokines in SLE, we measured the levels of IL-6, IL-8, IP-10, and IFN-I bioactivity in plasma as immunological factors in SLE patients and HDs. The plasma levels of these cytokines were significantly elevated in new-onset SLE patients (median: IL-6 new-onset SLE 114 pg ml^−1^, HD 100 pg ml^−1^, *P* = 0.0039; IL-8 new-onset SLE 142 pg ml^−1^, HD 110 pg ml^−1^, *P* = 0.0155; IP-10 new-onset SLE 2136 pg/ml^−1^, HD 117 pg ml^−1^, *P* = 0.0024) (Fig. 3A–C). The bioactivity of IFN-I in plasma was also elevated in new-onset and treated patients (median: new-onset SLE 0.582 O.D., HD 0.185 O.D., *P* = 0.0001) (Fig. 3D). The intensity of TLR9 expression slightly decreased on pDC in new-onset SLE patients (median: new-onset SLE 96.8, HD 117.5, *P* = 0.0757) (Fig. 3E), but its intensity did not change in other ICPs of new-onset patients compared with those in HDs (data not shown).

As a next step, we performed a Spearman’s correlation test between these immunological factors and the proliferative activity of each ICP (Fig. 3F). IL-6 was positively correlated with cMo (ρ = 0.466, *P* = 0.00038), eTreg (ρ = 0.444, *P* = 0.00078), PB (ρ = 0.486, *P* = 0.00019), and PC (ρ = 0.430, *P* = 0.00119). IL-8 was positively correlated with eTreg (ρ = 0.422, *P* = 0.00147). IP-10 was positively correlated with cMo (ρ = 0.507, *P* = 0.00001), dnCD3T (ρ = 0.506, *P* = 0.00001), CD8Tem (ρ = 0.497, *P* = 0.00013), CXCR5^−^ DNB (ρ = 0.455, *P* = 0.00054), and PB (ρ = 0.422, *P* = 0.00150). Bioactivity of IFN-I was positively correlated with cMo (ρ = 0.505, *P* = 0.00001), dnCD3T (ρ = 0.621, *P* < 0.00001), CD8Tem (ρ = 0.525, *P* = 0.00005), CD8Tcm (ρ = 0.524, *P* = 0.00005), Tph (ρ = 0.491, *P* = 0.00016), eTreg (ρ = 0.512, *P* = 0.00008), CXCR5^−^ nB (ρ = 0.489, *P* = 0.00017), CXCR5^−^ NSMB (ρ = 0.464, *P* = 0.00042), CXCR5^−^ SMB (ρ = 0.536, *P* = 0.00003), CXCR5^−^ DNB (ρ = 0.526, *P* = 0.00004), PB (ρ = 0.541, *P* = 0.00002), and PC (ρ = 0.477, *P* = 0.00027). The intensity of TLR9 in pDC was not significantly correlated with any ICP.

We also performed multiple regression analysis between the immunological factors and the proliferative activity of each ICP (Supplementary Table S3). The IL-8 level was positively correlated with the proliferative activity of eTreg (*P* = 0.0085). The IP-10 level was positively correlated with the proliferative activity of CXCR5^−^ nB (*P* = 0.0257). The bioactivity of IFN-I was positively correlated with the proliferative activity of CXCR5^−^ SMB (*P* = 0.0458) and PB (*P* = 0.0036). The intensity of TLR9 expression on pDC was negatively correlated with the proliferative activity of CD8Tem (*P* = 0.0047), CD8Tcm (*P* = 0.0124), and Tph (*P* = 0.0478). These results suggested that the proliferative activity level of some ICPs such as cMo, dnCD3T, Tph, eTreg, CXCR5^−^ nB, CXCR5^−^ SMB, CXCR5^−^ DNB, PB, and PC reflect the inflammatory cytokines in SLE pathology.

### Correlations between ICP proliferative activity and SLE symptoms

**Fig. 4.**
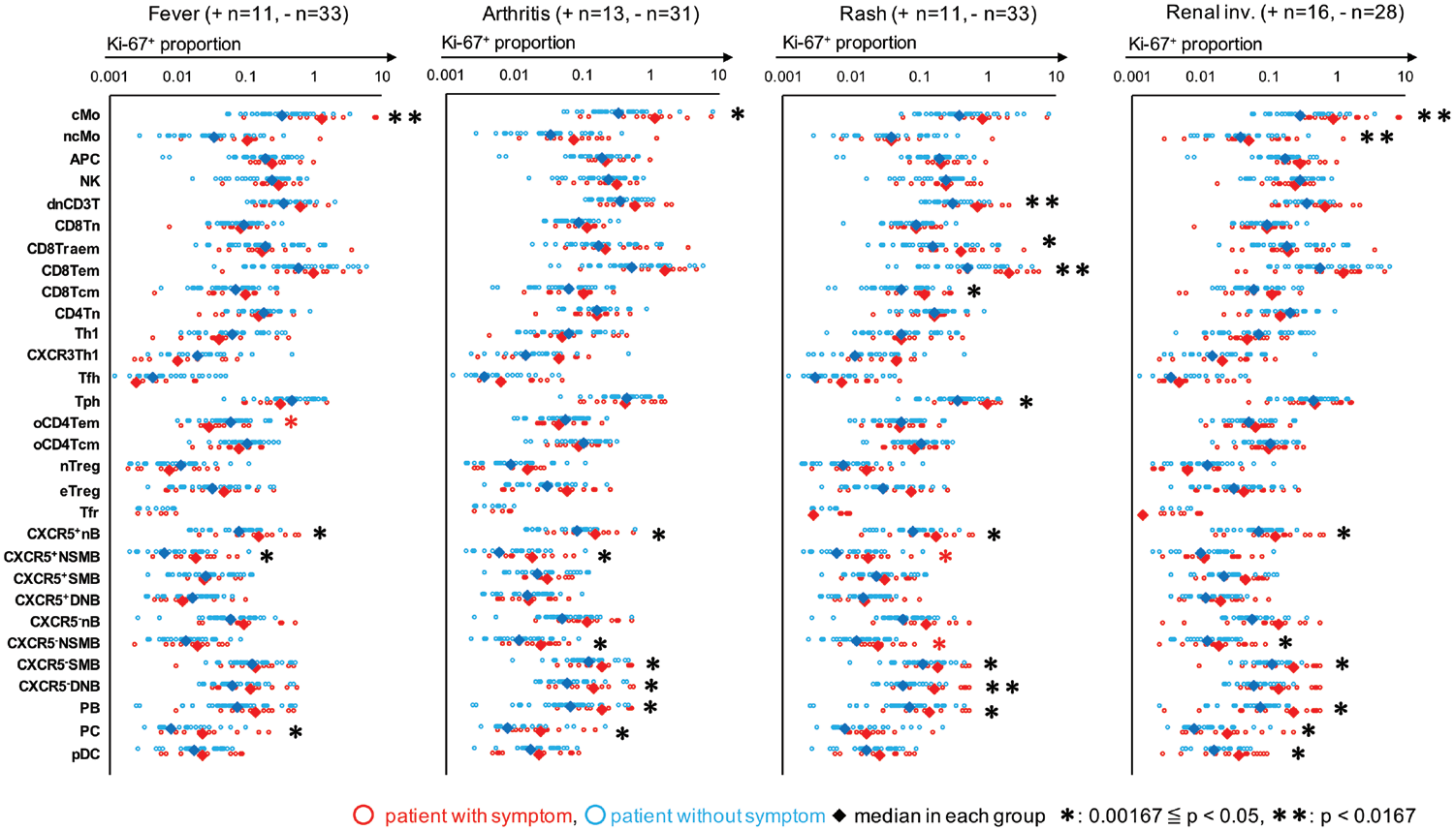
Correlation between ICP proliferative activity and SLE symptom. The correlation between the proliferative activity of each ICP in SLE patients and patients’ symptoms (arthritis, fever, skin rash, and renal involvement) was examined. The red circle indicates patients with each symptom, and the blue circle indicated patients without each symptom. The Mann–Whitney *U* test was used for comparisons between two groups. *0.00167 < *P* < 0.05, **P < 0.00167. See the Abbreviation list shown after Supplementary Table S4 for ICP abbreviations.

The proliferative activity of each ICP was compared in patients with or without specific clinical conditions (arthritis, fever, skin rash, renal involvement, serositis, and neural involvement) (Fig. 4). In patients with fever, the proliferative activity of cMo was significantly elevated (fever+:1.328%, fever‐: 0.343%, *P* = 0.00163). In patients with a skin rash, the proliferative activity of CD8Tem, dnCD3T, and CXCR5^−^ DNB were significantly elevated (rash+: 2.031%, rash‐: 0.512%, *P* = 0.00066; rash+: 0.710%, rash‐: 0.315%, *P* = 0.00035, and rash+: 0.167%, rash‐: 0.059%, *P* = 0.00083, respectively). In patients with renal involvement, the proliferative activity of cMo, APC, and CXCR5^−^ DNB was significantly elevated (rash+: 0.869%, rash‐: 0.280%, *P* = 0.00136; rash+: 0.282%, rash‐: 0.168%, *P* = 0.00112; and rash+: 0.138%, rash‐: 0.060%, *P* = 0.00083, respectively). In patients with arthritis, the proliferative activity was not significantly elevated in any ICPs and slightly increased in cMo, CXCR5^−^ DNB, PB, and PC (renal+: 1.142%, renal‐: 0.343%, *P* = 0.0069; renal+: 0.148%, renal‐: 0.059%, *P* = 0.0058; renal+: 0.191%, renal‐: 0.068%, *P* = 0.0031; and renal+: 0.024%, renal‐: 0.008%, *P* = 0.0032, respectively). In patients with serositis and neural involvement, the proliferative activity was not significantly elevated in any ICPs.

Logistic regression analysis based on the correlation of the proliferative activity of each ICP with specific conditions was performed (Supplementary Table S4). The proliferative activity of cMo was associated with the complication of fever (odds ratio: 18.3, *P* = 0.0358). The proliferative activity of CXCR5^−^ DNB slightly associated with the complication of rash (odds ratio: 8930, *P* = 0.0836). However, there were no ICPs associated with the complications of arthritis and renal involvement in this analysis. These results suggested that the proliferative activity level of cMo and CXCR5^−^ DNB reflect the clinical symptoms such as fever and rash in SLE pathology, respectively.

Whether the bioactivity of IFN-I is associated with SLE clinical conditions was also examined. SLE patients were divided into two groups: those with and without each condition and the intensity of IFN-I bioactivity in those groups was evaluated. However, there was no significant difference in the bioactivity of IFN-I between patients with a specific clinical condition and those without the condition (Supplementary Figure S5).

### Classification of SLE patients on the basis of ICP proliferative activity

**Fig. 5.**
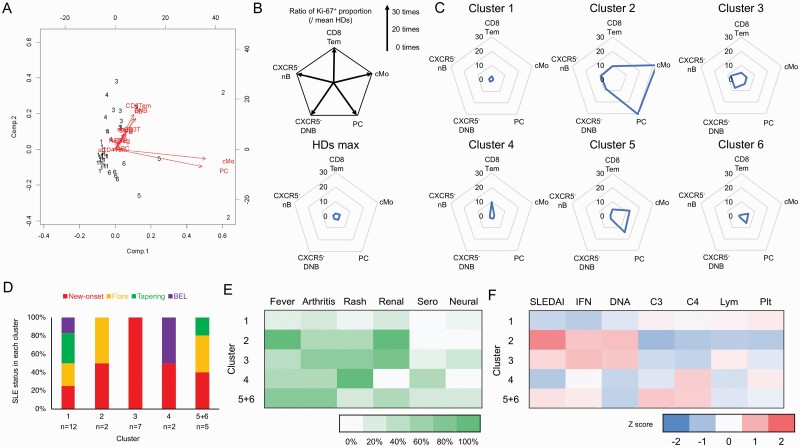
Classification of SLE patients on the basis of ICP proliferative activity. (A) *k*-Means clustering of SLE patients based on the proliferative activity of each ICP associated with SLE clinical phenotypes. Numbers in this figure indicate the individual cluster numbers. (B) The radar chart indicates proliferative activity status. Proliferative activity is shown as the ratio of the Ki-67^+^ proportion in each ICP between individual data of SLE patients and mean data of HDs. Maximum proliferative activity level in HDs is also shown. (C) The mean proliferative activity status in SLE patients classified into each of the six clusters is shown as radar charts. (D) Percentages of patients with new-onset (red), treated (orange), steroid-tapering (green), or belimumab-treated (purple) SLE in the six clusters are shown in a bar graph. Clusters 5 and 6 are regarded as the same populations with similar proliferative activity statuses. (E) The percentages of patients with each symptom or organ involvement (fever, arthritis, rash, renal involvement, serositis, and neural involvement) in each of the six clusters is shown as a heatmap. (F) Heatmap depicting *Z* scores of clinical findings (SLEDAI, IFN-I bioactivity, anti-DNA antibody titer, C3 level, C4 level, lymphocyte count, and platelet count) from blood tests. SLEDAI, Systemic Lupus Erythematosus Disease Activity Index; IFN, interferon; IFN-I, type I IFN; Lym, the number of lymphocytes; Plt, the number of platelets. See the Abbreviation list shown after Supplementary Table S4 for ICP abbreviations.

On the basis of the proliferative activity of each ICP, we stratified SLE patients by *k*-means clustering analysis. In *k*-means clustering analysis, the information of the Ki-67^+^ proportions of 12 ICPs (cMo, APC, dnCD3T, CD8Tem, oCD4Tcm, eTreg, CXCR5^−^ nB, CXCR5^−^ NSMB, CXCR5^−^ SMB, CXCR5^−^ DNB, PB, and PC) that correlated with SLE clinical phenotype were used (Fig. 5A). The proliferative activity of the 12 ICPs was divided into two components. In component 1, proliferative activities of cMo and PC were major contributors. For component 2, the proliferative activities of CD8Tem, CXCR5^−^ nB, and CXCR5^−^ DNB were major contributors. According to proliferative activities, cMo, PC, CD8Tem, CXCR5^−^ nB, and CXCR5^−^ DNB were chosen as important factors for classification, and patients were organized into six clusters in the first step.

To visualize the characteristics of these six patient groups, radar charts were produced (Fig. 5B). The proliferative activity was shown as the rate of Ki-67^+^ proportion of each ICP between individual data and mean data of HDs. At first, the proliferative activity status in HD was evaluated. The maximum proliferative activity in the five ICPs in HDs was within five times the average HD proliferative activity (the mean ratio of the Ki-67^+^ proportion: CD8Tem 1.873, cMo 2.953, PC 2.972, CXCR5^−^ nB 1.911, and CXCR5^−^ DNB 2.023). The proliferative activity status of six SLE patient clusters is shown (Fig. 5C). For patients in cluster 2, the proliferative level of all five ICPs were higher, especially for cMo and PC, which were remarkably high (the mean ratio of Ki-67^+^ proportion: CD8Tem 9.258, cMo 32.795, PCs 30.383, CXCR5^−^ nB 8.729, and CXCR5^−^ DNB 8.264). In contrast, for cluster 1 patients, the proliferation level of all five ICPs were low (the mean ratio of the Ki-67^+^ proportion: CD8Tem 2.232, cMo 1.343, PC 1.214, CXCR5^−^ nB 1.792, and CXCR5^−^ DNB 1.817). In cluster 3, the proliferation level of CXCR5^−^ nB, and CXCR5^−^ DNB were high (the mean ratio of the proportion of immune cells expressing high levels of Ki-67: CD8Tem 4.806, cMo 4.153, PC 4.011, CXCR5^−^ nB 7.776, and CXCR5^−^ DNB 7.473). Patients in cluster 4 only possessed high CD8Tem proliferation levels (the mean ratio of the Ki-67^+^ proportion: CD8Tem 9.893, cMo 2.082, PC 2.122, CXCR5^−^ nB 1.185, and CXCR5^−^ DNB 1.592). In clusters 5 and 6, the proliferation levels of both cMo and PC on the higher side (the mean ratio of the proportion of Ki-67^+^ cells: CD8Tem 4.758, cMo 12.740, PC 14.212, CXCR5^−^ nB 2.043, and CXCR5^−^ DNB 1.539; CD8Tem 1.116, cMo 5.382, PC 6.070, CXCR5^−^ nB 1.528, and CXCR5^−^ DNB 1.260, respectively). Following these results, we regarded clusters 5 and 6 as populations with similar proliferative activity statuses.

To assess whether ICPs proliferative activity clusters reflected a specific SLE phenotype, we evaluated clinical findings (SLEDAI, anti-DNA antibody titer, C3 and C4 levels, and Pt count), immunological factors (IL-6, IL-8, IL-10, the bioactivity of IFN-I, and TLR9 expression), and clinical symptoms (fever, arthritis, rash, and renal involvement) in each cluster. The percentages of new-onset SLE patients, flared SLE patients, and steroid-tapered SLE patients in each cluster were shown (Fig. 5D). In cluster 3, all patients were new-onset SLE. Steroid-tapered SLE patients were slightly more included in clusters 1 and 5 + 6. SLE patients who started belimumab seemed be included more in clusters 1 and 4. The percentages of clinical conditions and *Z* score of clinical findings of SLE patients in each cluster were evaluated (Fig. 5E and F). The clinical SLE phenotypes in individual patients classified into the six clusters are shown in Supplementary Figure S6. In cluster 1, the SLE clinical conditions complication rate was low. In cluster 2, the complication rates of fever and renal involvement were both 100%. In cluster 3, the complication rates of arthritis, rash, and renal involvement were 71.4%, 71.4%, and 85.7%. In cluster 4, there was a 100% complication rate for rash. In cluster 5, the complication rates of fever and arthritis were both 75% (Fig. 5E). The *Z* score of SLEDAI was high in cluster 2 (1.57), but not in cluster 1 (‐0.59) (Fig. 5F). The IFN-I bioactivity *Z* score was high in cluster 2 (0.75) and 3 (0.86), but not in cluster 1 (‐0.75). The *Z* score of anti-DNA antibody titer was high in cluster 2 (0.85) and 3 (0.75), but not cluster 4 (‐0.58) and 5 + 6 (‐0.65). The C3 level *Z* score was low in cluster 2 (‐1.08), but not cluster 5 + 6 (0.73). The *Z* score of C4 level was low in cluster 2 (‐0.83) and 3 (‐0.63), but not cluster 4 (0.66) and 5 + 6 (0.65). The *Z* score of lymphocyte count was low in cluster 2 (‐0.75), but not cluster 3 (0.24). The *Z* score of platelet count was low in cluster 2 (‐0.78), but not in cluster 4 (0.26) (Fig. 5F). These results suggest that the proliferation status may reflect SLE phenotype and be a useful marker for SLE patient classification.

### ICP proliferative activity dynamics in SLE patients before and after immunosuppressive treatment

**Fig. 6.**
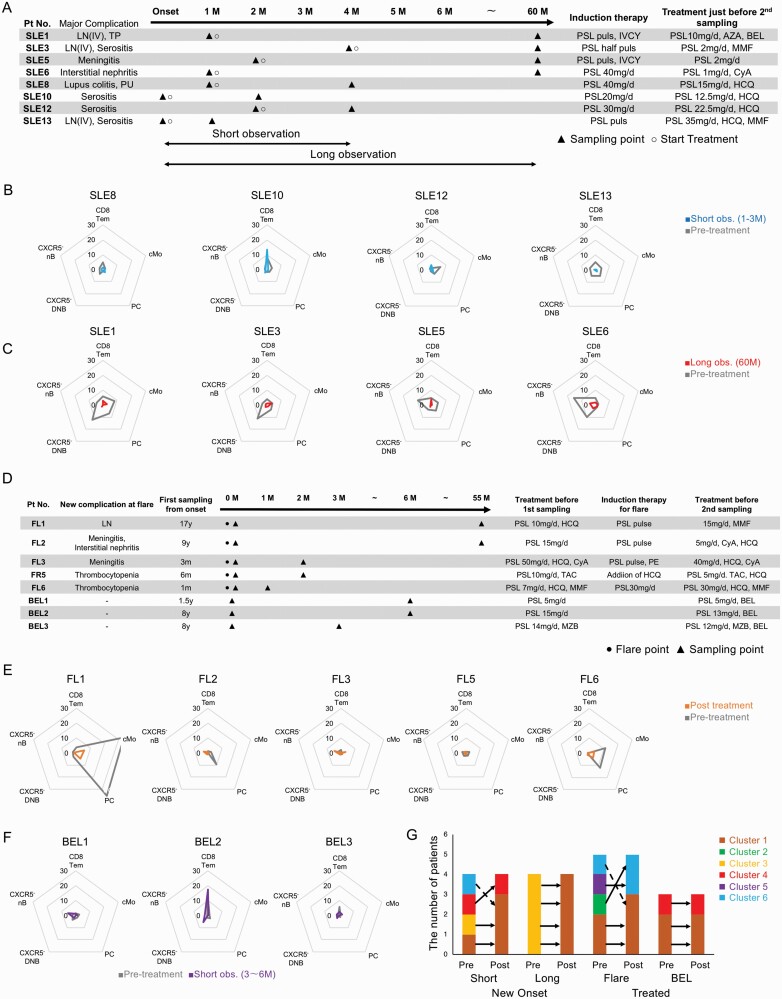
ICP proliferative activity dynamics in SLE patients before and after immunosuppressive treatment. (A, D) The clinical course and time point of blood sampling in patients with new-onset SLE (A) and treated SLE (D) are shown. (B) Proliferative activity statuses in four patients with new-onset SLE before induction therapy (gray) and at 1–3 months after induction therapy (blue) are shown. (C) The proliferative activity statuses in four patients with new-onset SLE before induction therapy (gray) and at 60 months after induction therapy (red) are shown. (E) The proliferative activity statuses in five patients with flared SLE before (gray) and after treatment (orange) are shown. (F) The proliferative activity statuses in three patients with treated SLE before (gray) and after belimumab treatment (purple) are shown. (G) Changes in each proliferative activity status pattern (cluster) in individual SLE patients are shown. The arrow with the solid line indicated patients with a short observation period, and the arrow with the broken line indicated patients with a long observation period. M, month(s); LN, lupus nephritis; TP, thrombocytopenia; PU, proteinuria; PSL, prednisolone; IVCY, intravenous cyclophosphamide; AZA, azathioprine; BEL, belimumab; MMF, mycophenolate mofetil; CyA, cyclosporine A; HCQ, hydroxychloroquine; obs, observation; y, year(s); TAC, tacrolimus; MZB, mizoribine; PE, plasma exchange. See the Abbreviation list shown after Supplementary Table S4 for ICP abbreviations.

To assess the influence of immunosuppressive treatments on SLE patients’ ICP proliferative activity, proliferative activities for cMo, PC, CD8Tem, CXCR5^−^ nB, and CXCR5^−^ DNB were determined before and after treatment. The blood sampling strategy for patients with new-onset SLE is shown in Fig. 6A. These data were converted to radar charts. Over short observation (1–3 months post-induction therapy), the proliferative activity statuses of three patients shifted to cluster 1, but the status of one patient (SLE10) remained in cluster 4 (Fig. 6B). During the long-term observation (5 years after induction therapy), the proliferative activity status of four patients shifted to cluster 1 (Fig. 6C).

A second blood sample was drawn after induction therapy in five patients with flared SLE, and in three treated SLE patients after belimumab therapy. The blood sampling strategy in treated SLE patients is shown in Fig. 6D. The proliferative activity statuses of three patients (FL2, FL3, and FL4) had shifted to cluster 1. In the patient with flared SLE exhibiting lupus nephritis (FL1), the proliferative activity status shifted from cluster 2 to cluster 6, and in the patient with flared SLE exhibiting thrombocytopenia (FL6), the proliferative activity shifted from cluster 5 to cluster 6 (Fig. 6E). The proliferative activity status in three patients who started belimumab therapy did not change post-treatment (Fig. 6F). According to these results from 16 patients with new-onset SLE, flared SLE, SLE with belimumab, and steroid-tapered SLE, proliferative activity status, after immunosuppressive treatment, clusters changed to clusters 1, 4, and 6 (Fig. 6G).

In four of the five patients with steroid-tapered SLE (ST1-5), proliferative activity statuses were grouped in cluster 1. However, the SLE ST3 proliferative activity status was cluster 5; nevertheless, the clinical findings were similar among these five patients (Supplementary Figure S7). These results showed that, despite immunosuppressive treatments, for some SLE patients, the proliferative activity of some ICPs remain.

## Discussion

For SLE patients, there is no clarification of ICPs cellular activity status, and no consensus provided on the proliferative activity status of ICPs. Therefore, SLE clinical practice has not used information related to patients’ immune cell activity. In this study, we analyzed PBMCs of SLE patients using mass cytometry and identified a wide range of ICPs. Proliferative activity was estimated by determining the proportion of cells expressing specific markers and Ki-67 among CD45^+^ cells (Ki-67^+^ proportion). The proliferative activities of ICPs such as cMo, CD8Tem, CXCR5^−^ nB, CXCR5^−^ DNB, and PC correlated with clinical findings (SLEDAI, anti-DNA antibody, C3, C4, and platelet counts), immunological factors (IL-6, IL-8, IL-10, the bioactivity of IFN-I, and TLR9 expression), and clinical symptoms (fever, arthritis, rash, and renal involvement). Thus, this approach successfully uncovered IPCs immunodynamics not easily recognized by ICP counting alone. For example, in SLE patients compared with HDs, proportions of cMo and PC among CD45^+^ cells were reduced, however their proliferative activities were markedly increased. To understand the immunokinetics of SLE, it is important to assess cellular activity, like proliferative activity, rather than just the general ICP proportions in peripheral blood. Furthermore, we attempted to stratify SLE patients based on the proliferative activity of five ICPs in association with disease activity. Disease activity was evaluated by the levels in clinical findings, immunological factors, and clinical symptoms. From this, SLE patients were classified into five clusters. Cluster 1 showed low proliferative activity in all five ICPs and low SLE disease activity. Patients in cluster 2 showed high proliferative activity in cMo and PC, as well as high disease activity including clinical findings (SLEDAI, and anti-DNA antibody), immunological factors (bioactive INF-I and anti-DNA antibody), and symptoms (fever and renal involvement) (Fig. 5E and F). Cluster 3 showed high proliferative activity in CXCR5^−^ nB and CXCR5^−^ DNB with renal involvement and moderate SLE disease activity. Cluster 4 showed high CD8Tem proliferative activity accompanied by rash and low SLE disease activity, and finally, cluster 5 + 6 showed high proliferative activity in cMo and PC with fever and mild SLE disease activity. After immunosuppressive treatments, in many patients, proliferative activity status declined, reflecting the use of this approach in evaluating anti-SLE drugs. For new-onset patients, after 1–3 months of induction therapy, patients classified into cluster 3 and cluster 6 converted into less active cluster 1. One cluster 2 patient (FL1) with flared SLE exhibiting lupus nephritis, after treatment, shifted from the active cluster 2 to the less active cluster 6. After 5 years of long-term observation after induction therapy, all patients in cluster 3 converted to the less active cluster 1, suggesting that anti-SLE drugs reduce ICPs proliferative activity (Fig. 6G). Cluster 4, with high CD8Tcm proliferative activity, was considered resistant to anti-SLE drugs including prednisolone (PSL). In patients with the flared disease, those in cluster 4 did not change cluster, even if treated with belimumab, a monoclonal antibody against B cell activating factor (BAFF). In some patients, a high CD8Tem proliferative activity level may predict unfavorable responses in patients to induction therapy and belimumab.

Anti-DNA antibody titers correlated positively with proliferative activity of 5 ICPs (cMo, eTreg, CXCR5^−^ nB, CXCR5^−^ DNB, and PC). The correlation between CXCR5^−^, but not CXCR5^+^, B cells and anti-DNA antibody titers might reflect extrafollicular antibody production by PC during SLE pathogenesis. Although C4 was negatively correlated with the proliferative activity of eTreg, CXCR5^−^ nB, and CXCR5^−^ DNB, C3 did not correlate with any immune cell populations proliferative activity. However, C3 consumption must influence SLE immune response, and it may be necessary to evaluate the association between C3 and ICPs using cellular activity markers other than proliferative activity.

Monocytes were identified and characterized by surface marker CD14 and CD16 expression. In SLE pathology, monocyte apoptosis was promoted by IFN-I, reducing the proportion of cMo (15). Monocytes in SLE patients were likely to enhance CD40L expression and promote B cell activation (32). Also, macrophages have shown a reduced ability to clear apoptotic debris and increased inflammatory cytokine production (2). In this study, the proportion of both cMo, and ncMo, were decreased in SLE patients compared with HDs. Inversely, however, proliferative activity was significantly elevated in cMo. There were patients with high proliferative activity of both cMo and PC. The proliferative activity of cMo may be associated with fever and kidney involvement. Thus, cMo-targeting strategies may be important for SLE therapeutics.

The influence of CD8^+^ T cells in SLE is not extensively investigated as their role and pathological significance are unclear. Previous reports indicated a reduction in CD8^+^ T cell cytolytic activity, and this may be associated with SLE patients’ susceptibility to infection (33). Effector CD8^+^ T cells were found in damaged renal tissue, suggesting an association with tissue injury (34). IL-10-producing CD28^−^ CD8^+^ T cells were also depleted in SLE patients, a possible contributor to decreased autoreactive immune response regulation (35). In this study, the CD8^+^ T cells proliferative activities, including CD8Traem, CD8Tem, and CD8cm, were found to be elevated compared to HD, despite no significant differences in ICP proportion (Fig. 1). Furthermore, CD8Tem proliferative activity was strongly associated with skin rash. Thus, it was included in the selected ICPs to build radar charts for SLE patients (Fig. 5B). In some patients with high CD8Tem proliferative activity, this activity remained even after treatment. We did not evaluate perforin or granzyme expression and could not assess the actual CD8^+^ T cell cytotoxic activity. In the future, CD8^+^ T cells should be investigated to elucidate the influence of CD8^+^ T cells on SLE pathogenesis.

B cells are an important ICP, involved in SLE pathogenesis, but few reports focus on B cell proliferative activity in SLE. Our results showed the proliferative activity of CXCR5^+^ B cells was not increased, but activities of CXCR5^−^ B cells, especially CXCR5^−^ nB and CXCR5^−^ DNB, were significantly increased. Therefore, we hypothesized that the SLE pathological significance of CXCR5^−^ B cells may be higher than CXCR5^+^ B cells. Furthermore, SLE patients with high proliferative CXCR5^−^ B cell activity, were more likely to have high disease activity, renal involvement, arthritis, and skin rash. IFN-I, IL-21 produced by Tph, TLR7 signaling, and BAFF is reportedly involved in extrafollicular B cell formation and goblet center-independent class switching. Extrafollicular nB rapidly differentiates into CXCR5^−^ DNB and further into antibody-producing cells by TLR7 signaling and IL-21 (36, 37). Antibody production by extrafollicular B cells is considered important in the initial infection response. During infection, short-lived PCs are induced by this pathway but long-surviving PC-produced autoantibodies may be induced in the pathology of SLE (38).

The association between IFN-I and the proliferative activity of each ICP was evaluated in this analysis. Most ICPs considered strongly associated with the SLE phenotype correlated positively with IFN-I. Clustering analysis for SLE patients showed slightly decreased IFN-I bioactivity of IFN-I in cluster 4 patients with high CD8Tem proliferative activity, and in cluster 5 + 6 patients with high proliferative activity of cMo and PC in comparison to clusters 2 and 3 patients with high CXCR5^−^ B cell proliferative activity. These results may be of interest in considering the immunopathology of patients with low IFN-I activity but residual SLE disease activity.

In this study, it was not possible to assess the number of Ki-67^+^ cells in 1 µl of blood from HDs. Therefore, accurately comparing the number of Ki-67^+^ cells between HDs and SLE patients was difficult. Our results showed in SLE patients, on average, a threefold higher number of Ki-67^+^ cells among CD45^+^ cells. In previous reports, lymphocyte counts were on average 0.5-fold lower in SLE patients (39). In view of this result, we consider that the percentage of Ki-67^+^ cells may also be remarkably elevated in SLE, although this is only speculation.

There is no clear consensus regarding the effect of freezing on Ki-67 protein stability. Histological diagnosis of malignant tumors by pathological evaluation using frozen sections of Ki-67-expressing cells is performed in clinical practice. In previous reports, Ki-67 expression was observed in lymphoma cells frozen at ‐150°C and stored for more than 100 weeks (40). It was also reported that Ki-67 protein in frozen melanoma cells was detected stably up to 60 min after thawing but not after 120 min (41). Studies have found both unchanged or decreased (~70%) proliferation of frozen cells compared to fresh cells (42, 43). Considering these reports, the proliferative ability of frozen cells appears maintained. Although an effect on Ki-67 expression following freezing cannot be denied, we believe, in this experiment, the effect was minimized as all samples were frozen and evaluated under similar experimental conditions.

There were some limitations to our study. The number of patients was small, and it was difficult to sufficiently adjust the patient background. Also, the small number of SLE patients with neuropsychiatric SLE (NPSLE) and serositis prevented sufficient analysis. In the clustering analysis, cluster 2 includes only two patients, and although they have in common the very high proliferative activity and disease activity of cMo and PC, the *k*-mean clustering plots are far apart (Fig. 5A). Further clustering analysis with more SLE patients may subdivide cluster 2. Observational periods after treatment were also not identical among patients as this was a retrospective study. Therapeutic drugs used also varied among the patients. Immune cell numbers used for analysis were lower in some ICPs. The proliferative activity of each ICP was assessed by the Ki-67^+^ ratio, but actual proliferative capacity was not assessed in *in vitro* experiments. Additionally, ICPs activation conditions were only evaluated by Ki-67 expression. The large number of ICP activity markers greatly complicates analysis, so to simplify this we focused on Ki-67. This study only assessed the proliferative activity of immune cells in peripheral blood, not those in tissues. Although we could stratify SLE patients into clusters using the Ki-67^+^ proportion among ICPs, it was not possible to assess the flare risk of SLE patients classified in each cluster (especially clusters 4 or 6).

In conclusion, immunophenotyping by mass cytometry revealed the dynamics of proliferative activity in peripheral ICPs linked to individual SLE patients’ clinical phenotypes (see the Graphical Abstract). SLE is still poorly understood with a high degree of patient heterogeneity, so there is a lack of evidence to determine clear therapeutic strategies. Establishing these multi-proliferative activity patterns as surrogates for phenotypes in heterogeneous SLE will aid in SLE patient stratification and selecting the appropriate therapeutic strategy. To achieve this, a detailed evaluation of ICPs activation in larger numbers of SLE patients is needed.

## Supplementary Material

dxac042_suppl_Supplementary_Figure_S1

dxac042_suppl_Supplementary_Figure_S2

dxac042_suppl_Supplementary_Figure_S3

dxac042_suppl_Supplementary_Figure_S4

dxac042_suppl_Supplementary_Figure_S5

dxac042_suppl_Supplementary_Figure_S6

dxac042_suppl_Supplementary_Figure_S7

dxac042_suppl_Supplementary_Tables
