# Supplementary figures and images for: The proliferative activity levels of each immune cell population evaluated by mass cytometry are linked to the clinical phenotypes of systemic lupus erythematosus
Source: Int Immunol. 2022 Aug 23;35(1):27–41. doi: 10.1093/intimm/dxac042 (PMC9860541; doi:10.1093/intimm/dxac042)

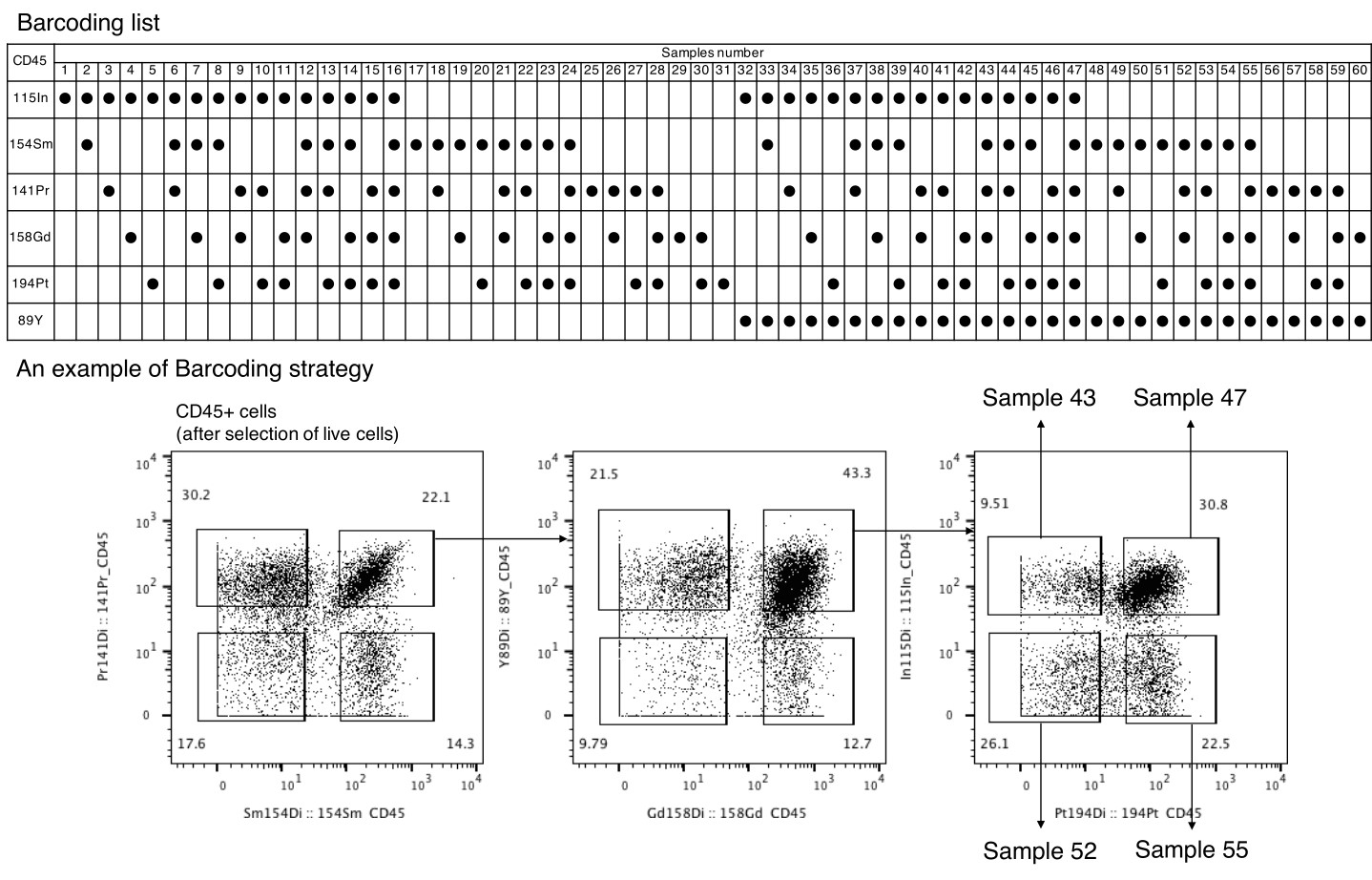

Supplement: dxac042_suppl_Supplementary_Figure_S1 [file dxac042_suppl_supplementary_figure_s1.jpeg]

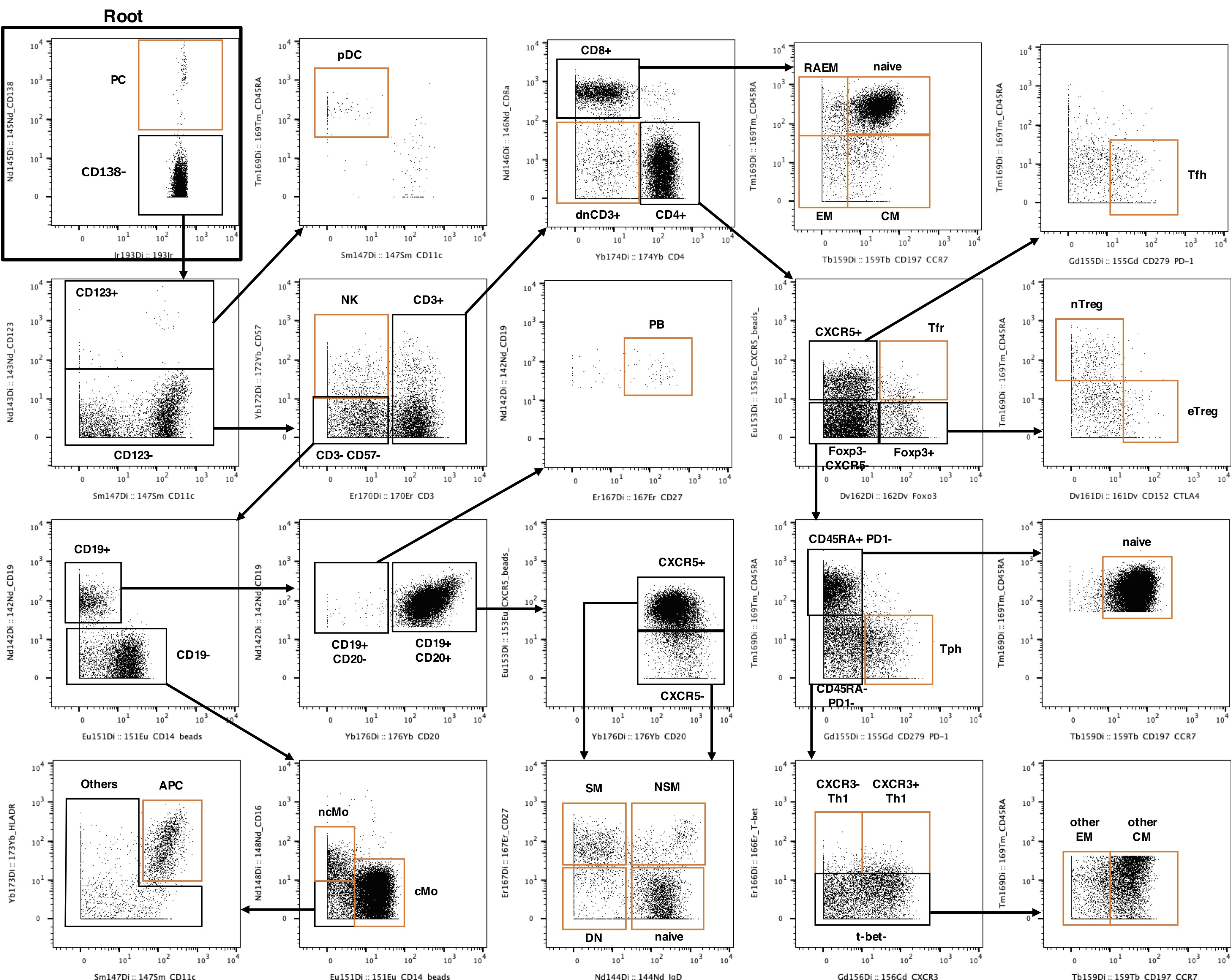

Supplement: dxac042_suppl_Supplementary_Figure_S2 [file dxac042_suppl_supplementary_figure_s2.jpeg]

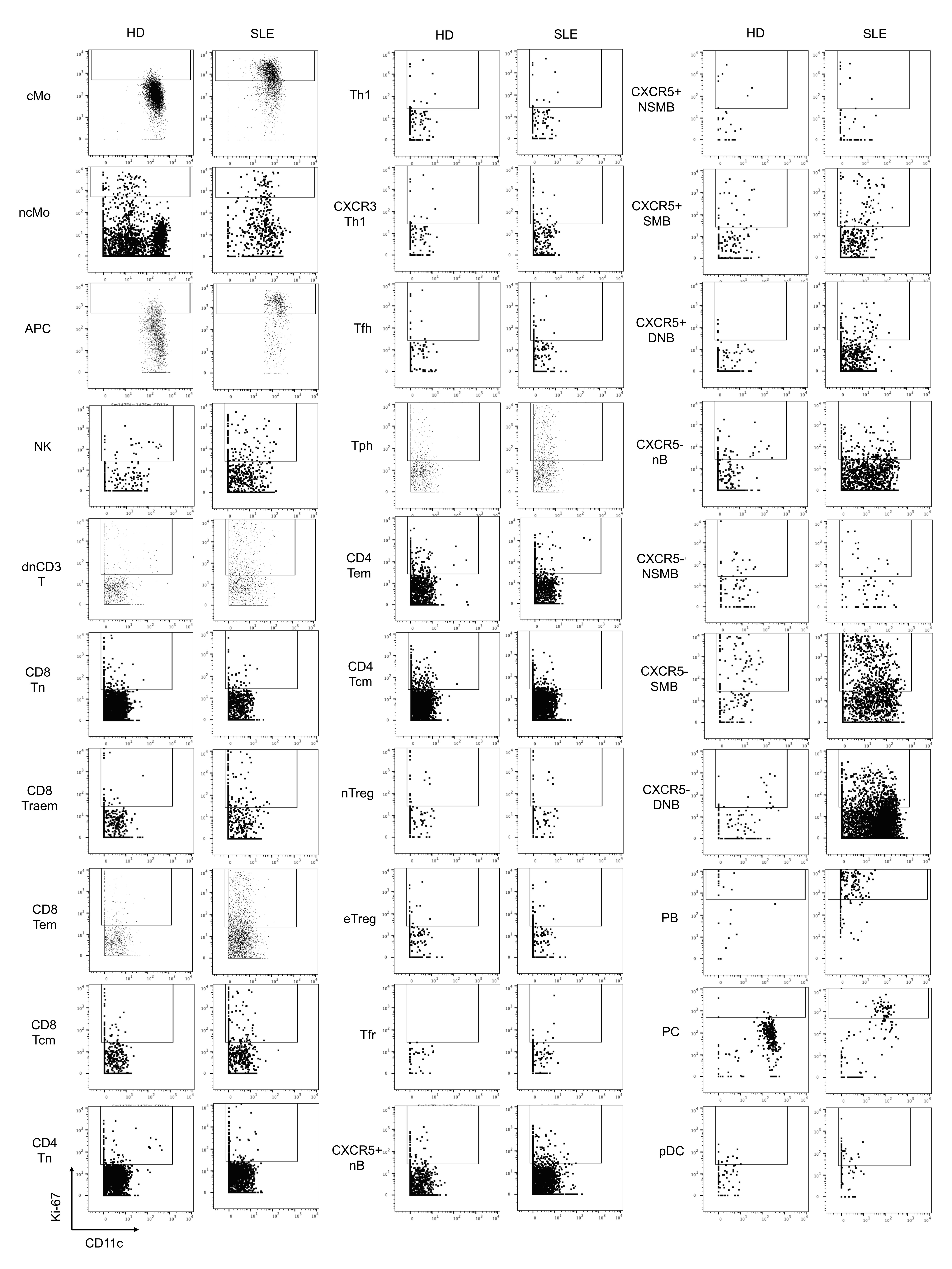

Supplement: dxac042_suppl_Supplementary_Figure_S3 [file dxac042_suppl_supplementary_figure_s3.jpeg]

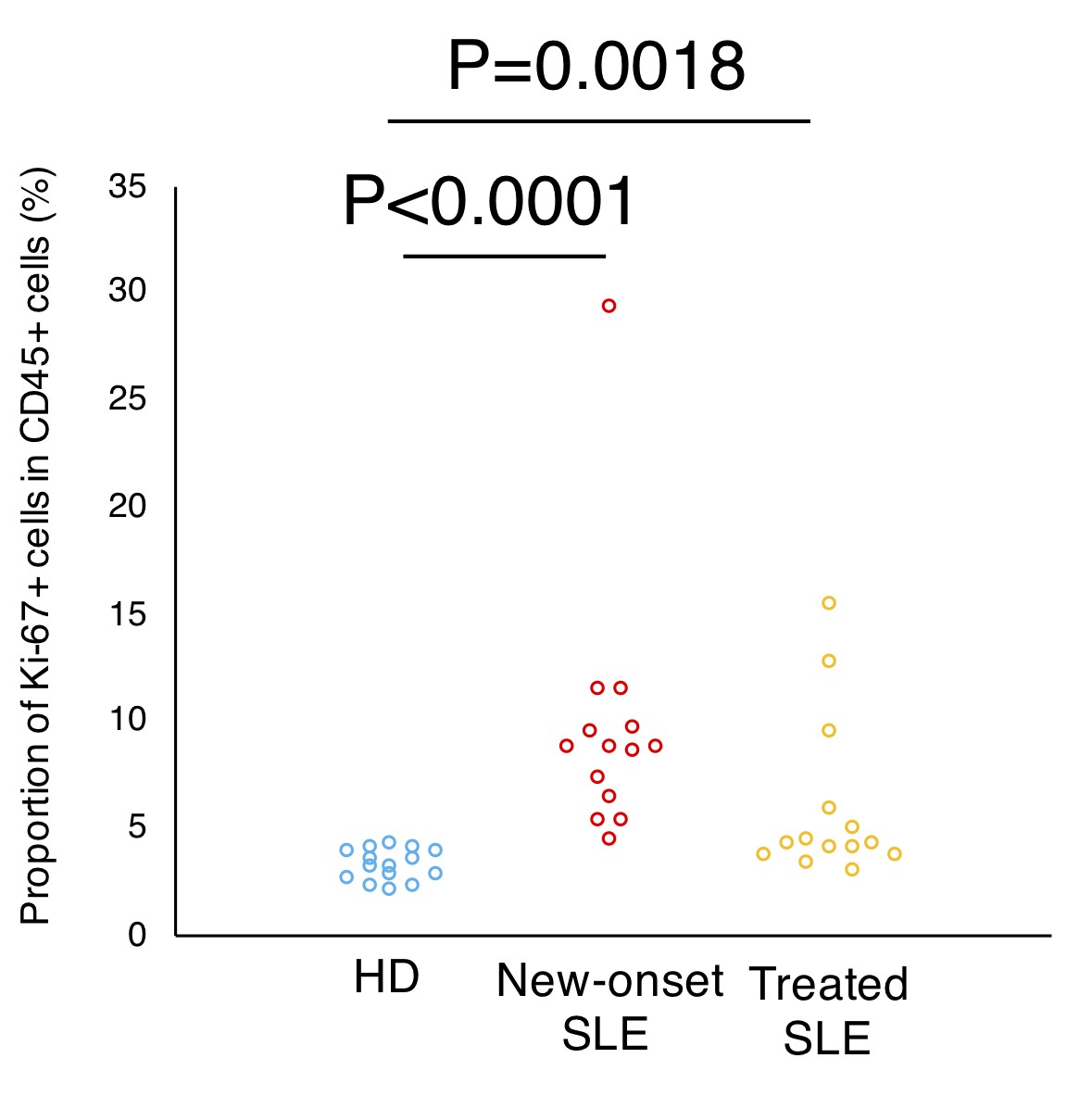

Supplement: dxac042_suppl_Supplementary_Figure_S4 [file dxac042_suppl_supplementary_figure_s4.jpeg]

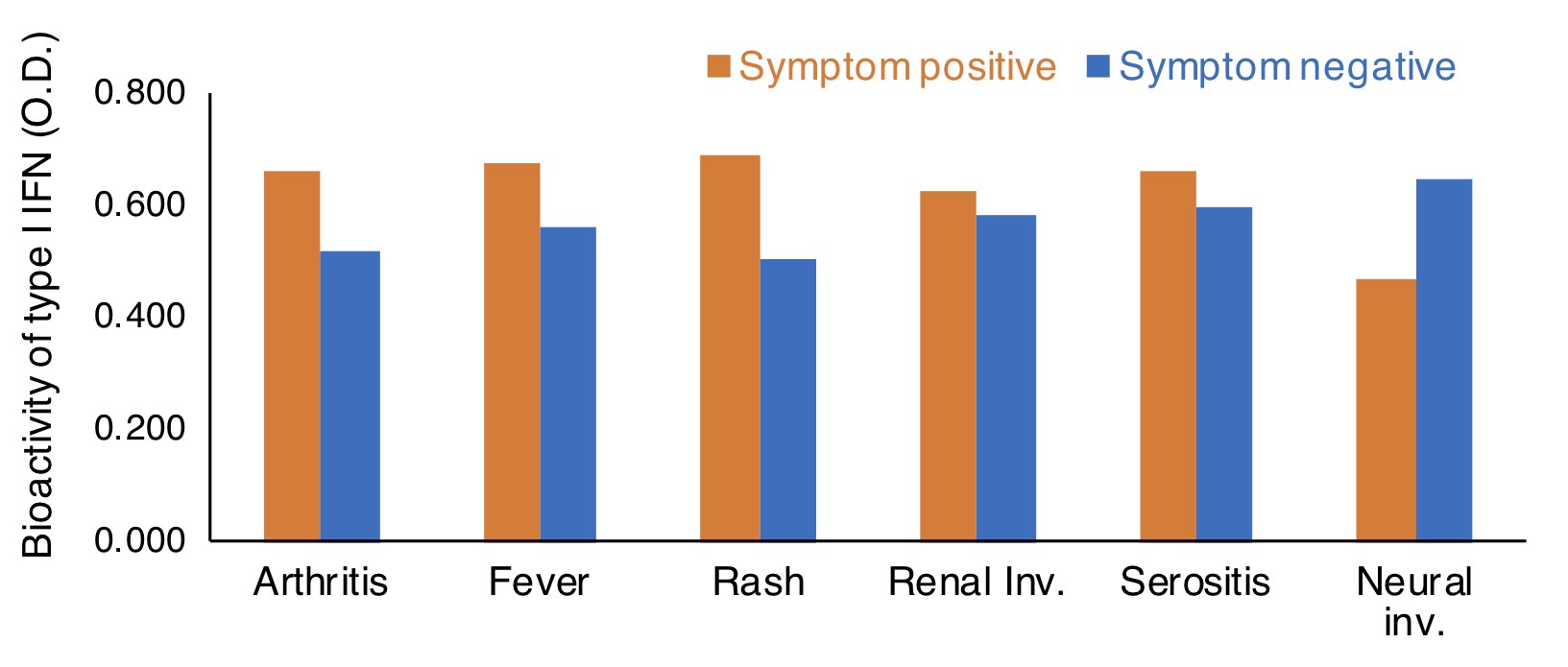

Supplement: dxac042_suppl_Supplementary_Figure_S5 [file dxac042_suppl_supplementary_figure_s5.jpeg]

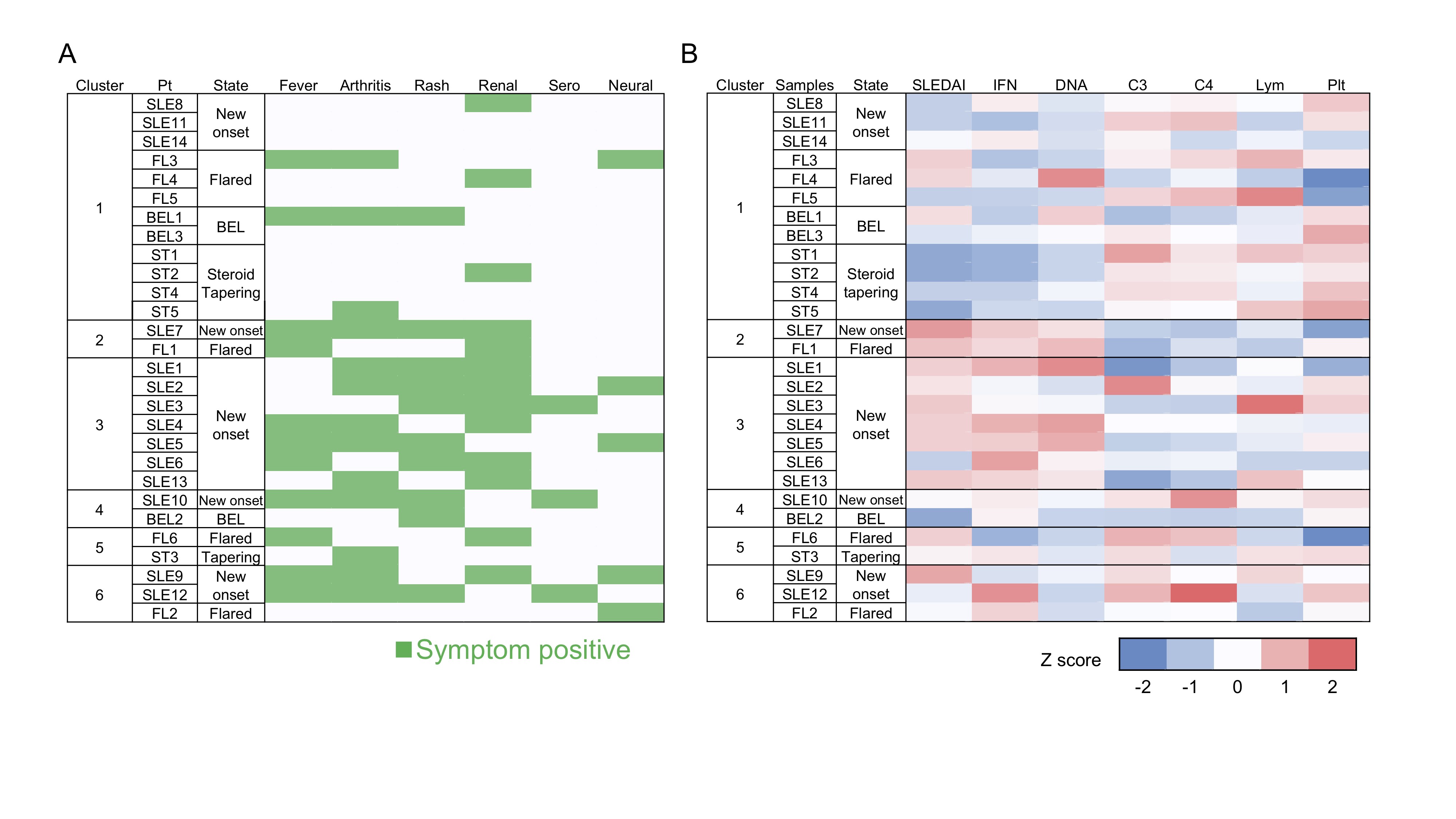

Supplement: dxac042_suppl_Supplementary_Figure_S6 [file dxac042_suppl_supplementary_figure_s6.jpeg]

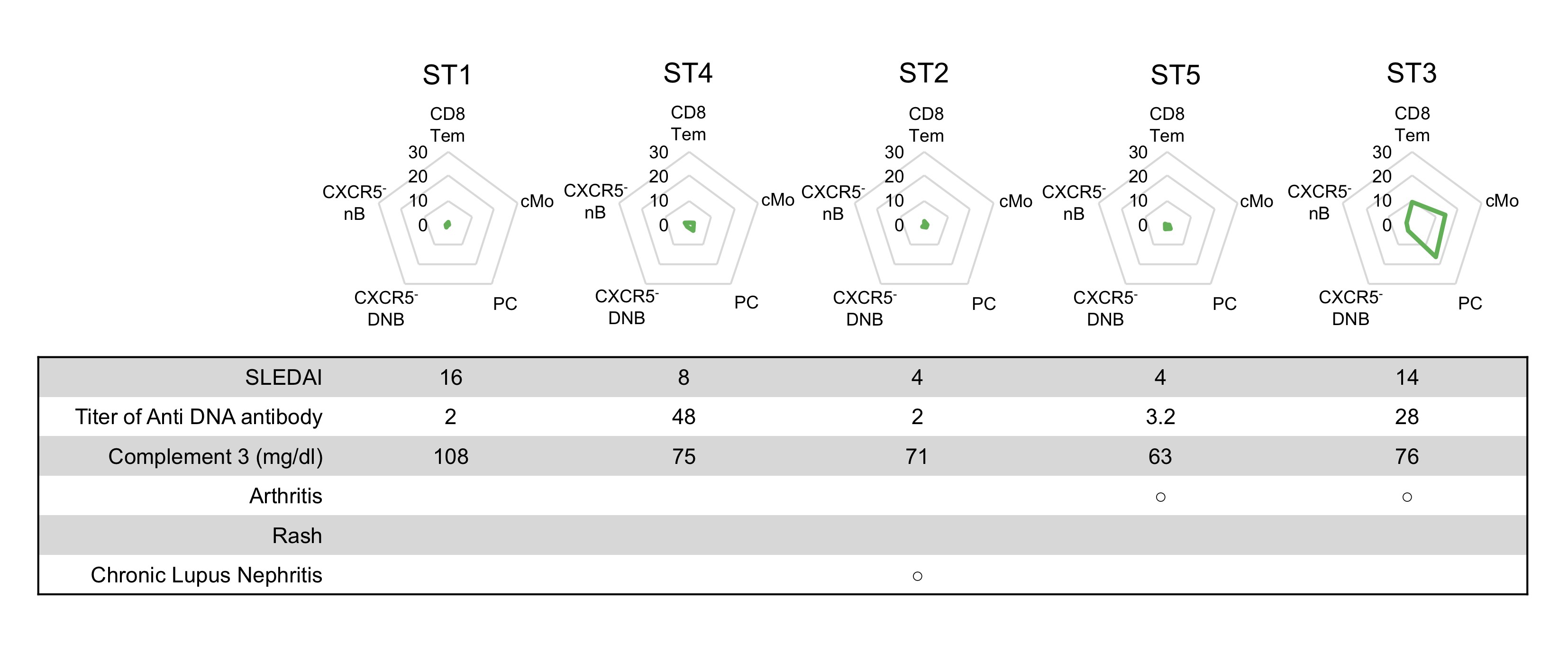

Supplement: dxac042_suppl_Supplementary_Figure_S7 [file dxac042_suppl_supplementary_figure_s7.jpeg]
