# Supplementary material for: The proliferative activity levels of each immune cell population evaluated by mass cytometry are linked to the clinical phenotypes of systemic lupus erythematosus
Source: Int Immunol. 2022 Aug 23;35(1):27–41. doi: 10.1093/intimm/dxac042 (PMC9860541; doi:10.1093/intimm/dxac042)
Supplement: dxac042_suppl_Supplementary_Tables [file dxac042_suppl_supplementary_tables.docx]

**SUPPLEMENTARY INFORMATION**

**Supplementary Table S1.** Information about the antibody panel for CyTOF.

| Isotype/metal | Target marker | Clone | Vender | Dilution |
| --- | --- | --- | --- | --- |
| 89Y | CD45 | HI30 | Fluidigm | 1:400 |
| 115In | CD45 | HI30 | BioLegend | 1:400 |
| 141Pr | CD45 | HI30 | BioLegend | 1:800 |
| 154Sm | CD45 | HI30 | Fluidigm | 1:400 |
| 158Gd | CD45 | HI30 | BioLegend | 1:800 |
| 194Pt | CD45 | HI30 | BioLegend | 1:800 |
| 142Nd | CD19 | HIB19 | Fluidigm | 1:100 |
| 143Nd | CD123 | 6H6 | Fluidigm | 1:100 |
| 144Nd | IgD | IA6-2 | BioLegend | 1:100 |
| 145Nd | CD138 | DL-101 | Fluidigm | 1:100 |
| 146Nd | CD8a | RPA-T8 | Fluidigm | 1:100 |
| 147Sm | CD11c | Bu15 | Fluidigm | 1:100 |
| 148Nd | CD16 | 3G8 | BioLegend | 1:100 |
| 149Sm | CD25 | 2A3 | Fluidigm | 1:100 |
| 151Eu | CD14 | M5E2 | Fluidigm | 1:100 |
| 152Sm | CD21 | Bu32 | BioLegend | 1:100 |
| 153Eu | CXCR5 | RF8B2 | Fluidigm | 1:100 |
| 155Gd | PD-1 | EH12.2H7 | Fluidigm | 1:100 |
| 156Gd | CXCR3 | G025H7 | Fluidigm | 1:100 |
| 159Tb | CCR7 | G043H7 | Fluidigm | 1:100 |
| 160Gd | FITC | FIT-22 | Fluidigm | 1:100 |
|  | TLR-7-FITC | 4G6 | NOVUS | 1:50 |
| 161Dy | CTLA-4 | 14D3 | Fluidigm | 1:100 |
| 162Dy | Foxp3 | 259D/C7 | Fluidigm | 1:100 |
| 163Dy | BAFF | T7-241 | Fluidigm | 1:100 |
| 164Dy | CXCL10 | IP-10 | R and D | 1:100 |
| 165Ho | PE | PE001 | Fluidigm | 1:100 |
|  | IFNa-PE | 7N4-1 | BD | 1:50 |
| 166Er | T-bet | 4B10 | BioLegend | 1:100 |
| 167Er | CD27 | O323 | BioLegend | 1:100 |
| 168Er | Ki-67 | Ki-67 | Fluidigm | 1:100 |
| 169Tm | CD45RA | HI100 | Fluidigm | 1:100 |
| 170Er | CD3 | UCHT1 | Fluidigm | 1:100 |
| 171Yb | TLR9 | S16013D | BioLegend | 1:100 |
| 172Yb | CD57 | HCD57 | Fluidigm | 1:100 |
| 173Yb | HLA-DR | L243 | Fluidigm | 1:100 |
| 174Yb | CD4 | SK3 | Fluidigm | 1:100 |
| 176Yb | CD20 | 2H7 | BioLegend | 1:100 |
| 209Bi | CD11b | ICRF44 | Fluidigm | 1:100 |

**Supplementary Table S2. Multiple regression analysis between clinical findings of SLE and Ki-67^+^ proportion of ICPs.**

| **Objective variable**  **(clinical findings)** | **Explanatory variable**  **(Ki-67^+^ proportion)** | **Regression coefficient estimates** | **P value** |
| --- | --- | --- | --- |
| SLEDAI  Anti-DNA antibody  C3 level  C4 level  Platelet count | cMo  PB  PC  cMo  DNB  eTreg  nB  PB  CXCR5^-^ DNB  eTreg  CXCR5- NSMB  Other CD4Tcm  CXCR5^-^ DNB  eTreg  CXCR5^-^ NSMB  APC  CXCR5^+^ nB  CXCR5^-^ nB | 0.5786  19.78  47.33  7.234  9.232  263.9  149.7  61.00  −36.68  −110.9  −29.29  122.1  −6.205  −39.10  −117.8  −20.06  −8.430  −5.038 | 0.7564  0.0037  0.5111  0.3171  0.9617  0.2317  0.2555  0.6942  0.2822  0.0438  0.8820  0.0033  0.6264  0.0604  0.1299  0.0034  0.5611  0.7485 |

**Supplementary Table S3. Multiple regression analysis between immunological factors of SLE and Ki-67^+^ proportion of ICPs.**

| **Objective variable**  **(Ki-67^+^ proportion)** | **Explanatory variable**  **(Immunological factors)** | **Regression coefficient estimates** | **P value** |
| --- | --- | --- | --- |
| cMo  CD8Tem  CD8Tcm  Tph  eTreg  CXCR5- nB  CXCR5- SMB  PB | IL-6  IL-8  IP-10  TLR9 (pDC)  IFN-I  IL-6  IL-8  IP-10  TLR9 (pDC)  IFN-I  IL-6  IL-8  IP-10  TLR9 (pDC)  IFN-I  IL-6  IL-8  IP-10  TLR9 (pDC)  IFN-I  IL-6  IL-8  IP-10  TLR9 (pDC)  IFN-I  IL-6  IL-8  IP-10  TLR9 (pDC)  IFN-I  IL-6  IL-8  IP-10  TLR9 (pDC)  IFN-I  IL-6  IL-8  IP-10  TLR9 (pDC)  IFN-I | 0.0186  −0.0011  −0.00008  0.0040  0.00391.427  −0.0036  −0.0077  0.0002  −0.0155  0.9578  −0.0001  −0.0004  <0.00001  −0.0007  0.0557  −0.0015  −0.0004  −0.00003  −0.0034  0.4221  −0.0002  0.0006  < −0.00001  −0.00004  0.1162  −0.0001  −0.0002  0.00003  −0.0006  0.0203  −0.0008  0.0008  −0.00001  −0.0005  0.2706  −0.0004  0.0003  −0.00001  −0.00003  0.1777 | 0.1815  0.8458  0.6435  0.4716  0.3293  0.7731  0.1286  0.3158  0.0047  0.5498  0.8359  0.1348  0.4985  0.0124  0.4462  0.7106  0.7578  0.6015  0.0478  0.3582  0.6712  0.0085  0.3473  0.8598  0.0624  0.3293  0.6371  0.0257  0.1896  0.8519  0.4601  0.7105  0.3278  0.3627  0.04579  0.3878  0.1737  0.1450  0.8802  0.0036 |

**Supplementary Table S4. Logistic regression analysis between each symptom of SLE and Ki-67^+^ proportion of ICPs.**

| **Objective variable**  **(symptoms)** | **Explanatory variable**  **(Ki-67^+^ proportion)** | **Odds ratio** | **P value** |
| --- | --- | --- | --- |
| Fever  Arthritis  Rash  Renal involvement | cMo  PC  PC  cMo  CXCR5^+^ nB  CXCR5^+^ NSMB  CXCR5^-^ DNB  CXCR5^-^ nB  CXCR5^-^ NSMB  PB  CD8Tem  dnCD3T  CXCR5^-^ DNB  PC  pDC  PB | 18.3  < 0.00001  < 0.00001  0.303  4.58  43.0  0.0217  6.39  2780  205  1.74  2.90  8930  1.000  1.030  1.000 | 0.0358  0.121  0.418  0.348  0.742  0.855  0.577  0.752  0.768  0.282  0.135  0.438  0.0836  0.179  0.098  0.420 |

**Abbreviation list for immune cell populations**

| Immune cell subsets | Abbreviations |
| --- | --- |
| CD14^+^ CD16^-^ classical monocyte | cMo |
| CD14^-^ CD16^+^ nonclassical monocyte | ncMo |
| CD14^-^ CD16^-^ CD11c^+^ HLA-DR^+^ antigen presenting cell | APC |
| CD57^+^ CD3^-^ natural killer cell | NK |
| CD4^-^ CD8^-^ double negative CD3^+^ T cell | dnCD3T |
| CD45RA^+^ CCR7^+^ naïve CD8^+^ T cell | CD8Tn |
| CD45RA^+^ CCR7^-^ CD8^+^ T cell | CD8Traem |
| CD45RA^-^ CCR7^-^ effector memory CD8^+^ T cell | CD8Tem |
| CD45^-^ CCR7^+^ central memory CD8^+^ T cell | CD8Tcm |
| CD45RA^+^ CCR7^+^ naïve CD4^+^ T cell | CD4Tn |
| t-bet^+^ type 1 helper CD4^+^ T cell | Th1 |
| CXCR3^+^ t-bet^+^ type 1 helper CD4^+^ T cell | CXCR3Th1 |
| CXCR5^+^ PD-1^+^ follicular helper CD4^+^ T cell | Tfh |
| CXCR5^+^ PD-1^+^ peripheral helper CD4^+^ T cell | Tph |
| Other central memory CD4^+^ T cell | oCD4Tcm |
| Other effector memory CD4^+^ T cell | oCD4Tem |
| CD45RA^+^ CTLA-4^-^ Foxp3^+^ naïve regulatory CD4^+^ T cell | nTreg |
| CD45RA^-^ CTLA-4^+^ Foxp3^+^ effector regulatory CD4^+^ T cell | eTreg |
| CXCR5^+^ PD-1^+^ Foxp3^+^ follicular regulatory T cell | Tfr |
| CXCR5^+^ IgD^+^ CD27^-^ naïve B cell | CXCR5^+^ nB |
| CXCR5^+^ IgD^+^ CD27^+^ nonswitched memory B cell | CXCR5^+^ NSMB |
| CXCR5^+^ IgD^-^ CD27^+^ switched memory B cell | CXCR5^+^ SMB |
| CXCR5^+^ IgD^-^ CD27^-^ double negative B cell | CXCR5^+^ DNB |
| CXCR5^-^ IgD^+^ CD27^-^ naïve B cell | CXCR5^-^ nB |
| CXCR5^-^ IgD^+^ CD27^+^ nonswitched memory B cell | CXCR5^-^ NSMB |
| CXCR5^-^ IgD^-^ CD27^+^ switched memory B cell | CXCR5^-^ SMB |
| CXCR5^-^ IgD^-^ CD27^-^ double negative B cell | CXCR5^-^ DNB |
| CD19^+^ CD20^-^ CD27^+^ plasma blast | PB |
| CD138^+^ plasma cell | PC |
| CD123^+^ plasmacytoid dendritic cell | pDC |

**Supplementary Figure Legends**

**Supplementary Figure S1.** Each patient’s FCS data were debarcoded by gating based on the staining patterns of the anti-CD45 antibody-conjugated metals.

**Supplementary Figure S2.** Gating strategy for immune cell populations (ICPs) in the peripheral blood. Each ICP with specific markers by gating on biaxial scatter plots using FlowJo 10.6.1.

**Supplementary Figure S3.** Gating for immune cells expressing Ki-67 in each ICP. In cMos, ncMos, APCs, PBs and PCs, the proportions of immune cells expressing high levels of Ki-67 were gated based on the comparison between SLE patients and HDs.

**Supplementary Figure S4**. The Ki-67^+^ cells among CD45^+^ cells in patients with new-onset, patients with treated SLE and HDs.

**Supplementary Figure S5.** The bioactivity of type I interferon (IFN-I) was compared between patients with new-onset SLE complicated with a specific symptom or organ involvement (orange; the numbers of patients with arthritis, fever, rash, renal involvement, serositis, or neural involvement were 9, 7, 8, 9, 3, and 3, respectively) and those without symptoms or organ involvement (blue).

**Supplementary Figure S6.** The Z score of clinical findings (left) and the positivity of symptoms (right) in each patient with SLE classified by the expression levels of Ki-67 in ICPs are shown as a heatmap. SLEDAI: Systemic Lupus Erythematosus Disease Activity Index, IFN: type I interferon, Lym: the number of lymphocytes, Plt: the number of platelets, Renal: renal involvement, Sero: serositis, Neural: neural involvement.

**Supplementary Figure S7.** The proliferative activity statuses in individual patients with steroid-tapered systemic lupus erythematosus (SLE) are shown as radar charts. SLEDAI: Systemic Lupus Erythematosus Disease Activity Index. Abbreviation list showed abbreviations of each ICP.
